# Homochiral Self‐Sorted and Emissive Ir^III^ Metallo‐Cryptophanes

**DOI:** 10.1002/chem.201701348

**Published:** 2017-04-20

**Authors:** Victoria E. Pritchard, Diego Rota Martir, Samuel Oldknow, Shumpei Kai, Shuichi Hiraoka, Nikki J. Cookson, Eli Zysman‐Colman, Michaele J. Hardie

**Affiliations:** ^1^ School of ChemistryUniversity of LeedsWoodhouse LaneLeedsLS2 9JTUK; ^2^ Organic Semiconductor CentreEaSTCHEM School of ChemistryUniversity of St AndrewsSt Andrews, FifeKY16 9STUK; ^3^ Department of Basic ScienceGraduate School of Arts and SciencesThe University of Tokyo3–8-1 Komaba, Meguro-kuTokyo153-8902Japan

**Keywords:** cage compounds, homochiral self-sorting, iridium, phosphorescence, supramolecular chemistry

## Abstract

The racemic ligands (±)‐tris(isonicotinoyl)‐cyclotriguaiacylene (L1), or (±)‐tris(4‐pyridyl‐methyl)‐cyclotriguaiacylene (L2) assemble with racemic (Λ,Δ)‐[Ir(ppy)_2_(MeCN)_2_]^+^, in which ppy=2‐phenylpyridinato, to form [{Ir(ppy)_2_}_3_(L)_2_]^3+^ metallo‐cryptophane cages. The crystal structure of [{Ir(ppy)_2_}_3_(L1)_2_]⋅3BF_4_ has *MM*‐ΛΛΛ and *PP*‐ΔΔΔ isomers, and homochiral self‐sorting occurs in solution, a process accelerated by a chiral guest. Self‐recognition between L1 and L2 within cages does not occur, and cages show very slow ligand exchange. Both cages are phosphorescent, with [{Ir(ppy)_2_}_3_(L2)_2_]^3+^ having enhanced and blue‐shifted emission when compared with [{Ir(ppy)_2_}_3_(L1)_2_]^3+^.

Metallo‐cages are discrete 3D‐coordination assemblies with a hollow interior and have applications as hosts and nanoscale vessels.^1^ They form through the self‐assembly of multidentate ligands with metals, or with metal complexes with controlled available coordination sites (“metallo‐tectons”). Luminescent metallo‐cages are known,^2^, ^3^, ^4^, ^5^, ^6^ with most examples exhibiting fluorescence‐active ligands,^2^ alongside rarer examples of cages with pendant metal‐complex emissive groups.^3^ There are very few examples of metallo‐cages constructed from inherently phosphorescent structural components.^4^, ^5^, ^6^ Cyclometalated Ir^III^ complexes bearing either two N‐donor ligands or one $\hat{NN}$ chelating ligand represent an important subclass of phosphorescent materials.^7^ Lusby and co‐workers reported the enantiopure Ir^III^ metallo‐cage [{Ir(ppy)_2_}_6_(tcb)_4_]⋅(OTf)_6_ (tcb=1,3,5‐tricyanobenzene),^4^ which self‐assembles, despite the inertness of the d^6^ Ir^III^ centre, as the *C*,*C‐cis‐N*,*N‐trans* arrangement of the ppy ligands has a *trans‐*labilising effect. The cage shows red‐shifted emission compared with a monomeric analogue, and enhanced photoluminescence quantum yields (*Φ*_PL_). To date, this is the only report of a 3D metallo‐cage that utilizes [Ir(ppy)_2_] as the sole metal centre, although mixed metal examples are known.^5^

Here, we report two metallo‐cages of the type [{Ir(ppy)_2_}_3_(L)_2_]^3+^, in which L is a chiral tripodal ligand related to the molecular host cyclotriveratrylene (CTV). [M(chelate)]_3_L_2_ cages with CTV‐type ligands are known as metallo‐cryptophanes, and most examples feature square planar metals.^8^ The [{Ir(ppy)_2_}_3_(L)_2_]^3+^ cages reported here show homochiral sorting on crystallization and in solution, and slow ligand exchange behaviour is observed.

Cages [{Ir(ppy)_2_}_3_(L1)_2_]^3+^ **1** and [{Ir(ppy)_2_}_3_(L2)_2_]^3+^ **2** are formed from nitromethane mixtures of (Λ,Δ)‐[Ir(ppy)_2_(MeCN)_2_]⋅X (X=PF_6_^−^, BF_4_^−^) and (±)‐L1 or (±)‐L2 in 3:2 stoichiometry (Scheme 1). Electrospray ionization mass spectrometry (ESI‐MS) gives a triply charged *m*/*z* peak at 983.1120 (cage **1**) or at 955.2853 (cage **2**), along with [{Ir(ppy)_2_}(L)]^3+^ and [{Ir(ppy)_2_}_2_(L)_2_]^3+^ fragment species (Figures S3 and S4 in the Supporting Information). Initial ^1^H NMR spectra of [Ir(ppy)_2_(NCMe)_2_]⋅X and L in [D_3_]‐MeNO_2_ show considerable broadening of the resonances and chemical shift changes, most saliently the ppy protons *ortho* to the coordinating N (H_A′_) and C (H_H′_) move upfield and downfield, respectively. For cage **2**, the previously sharp CH_2_ bridge singlet of L2 at 5.19 ppm becomes a complex multiplet as free rotation is hindered (Figure S15). ROESY spectra of **1** and **2** give the expected couplings, including between H_H′_ on the ppy ligands and the *ortho* pyridyl protons of L (Figures S8 and S16). Diffusion ordered NMR spectroscopy in [D_3_]‐MeNO_2_ for **1**⋅3PF_6_ (Figure S9) gave a hydrodynamic radius of 18.99 Å.

**Scheme 1:**
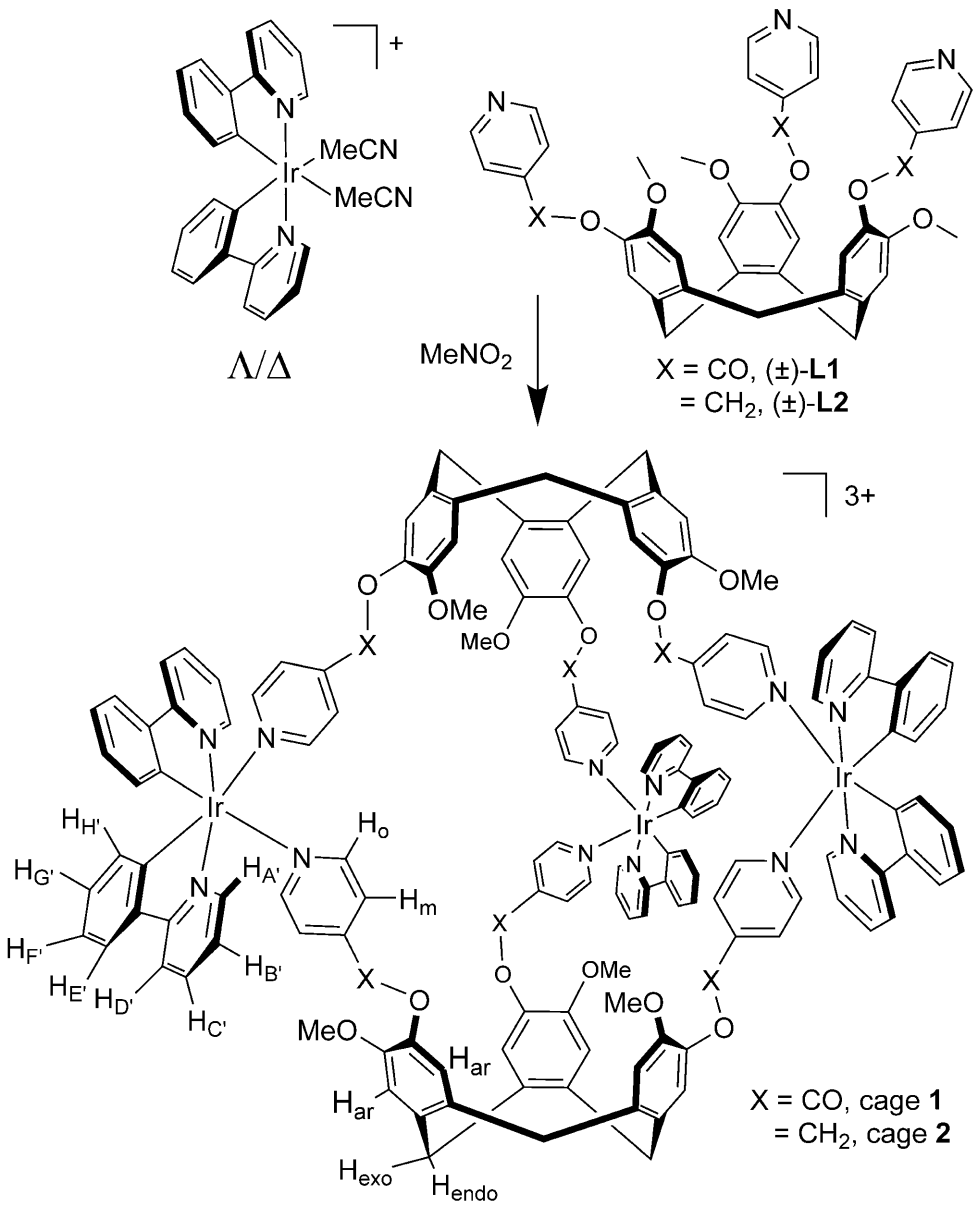
Synthesis of metallo‐cryptophane cage species.

The structure of **1**⋅3BF_4_⋅*n*(MeNO_2_) was confirmed by crystallography (Figure 1).^9^ There are two independent cage **1** cations that show minor structural differences. Anions and additional solvent were not located due to significant disorder. Each cage has three pseudo‐octahedrally coordinated Ir^III^ centres, each with two ppy ligands and the pyridyl groups from two L1 ligands are in a *cis* arrangement. The two L1 ligands bridge between three Ir^III^ centres. The average torsion angle between *cis*‐pyridyl groups is 38.04°, typical for [Ir(ppy)_2_(pyridyl)_2_]‐type complexes^10^ with the bowl shape of CTV‐type ligands being able to accommodate these torsion angles within the cage structure.

**Figure 1:**
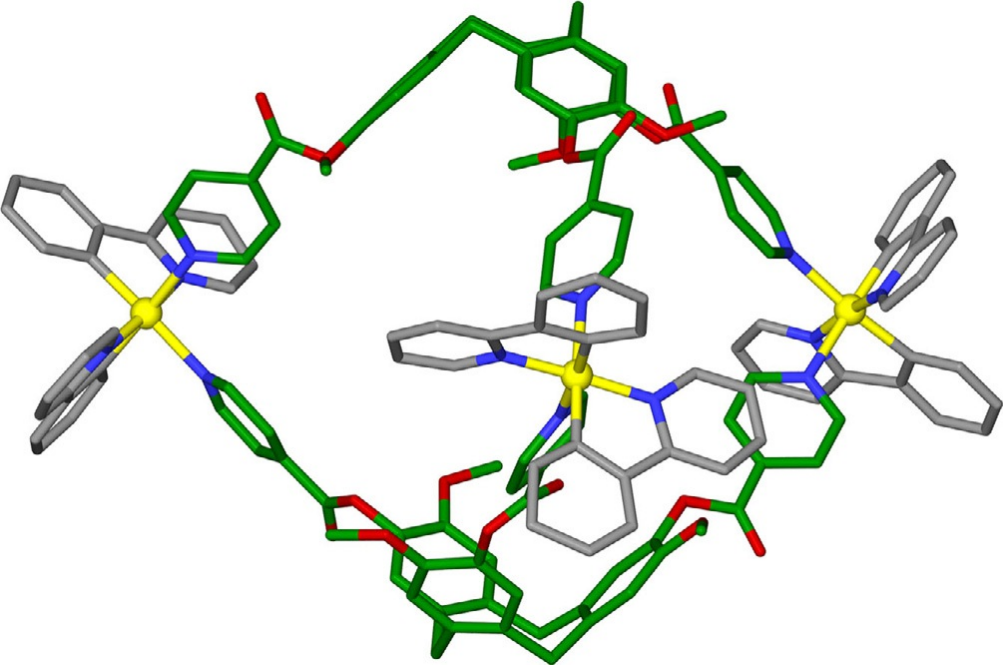
A [{Ir(ppy)_2_}_3_(L1)_2_]^3+^ cage from the crystal structure of **1**⋅3BF_4_⋅*n*(CH_3_NO_2_); L1 and ppy ligands shown in green and grey, respectively.

Both L1 ligands within each cage **1** are the same enantiomer, giving the chiral *anti*‐cryptophane isomer. Each [Ir(ppy)_2_] unit within a cage has the same chirality, such that only the enantiomeric *MM*‐ΛΛΛ and *PP*‐ΔΔΔ cage isomers are observed in the structure. Given that the Λ and Δ enantiomers of the [Ir(ppy)_2_]^+^ moieties and the *M* and *P* enantiomers of the l‐types ligands are present in the reaction mixture, there are twelve possible stereoisomers of the cage. The ^1^H NMR spectra of both cages **1** and **2** undergo significant sharpening upon standing (Figures S7 and S15 in the Supporting Information), and fully equilibrate after several months. The ^1^H NMR spectrum of cage **1**⋅3PF_6_, collected after 3 months of standing, is virtually identical to that of the single crystals of **1**⋅3BF_4_⋅*n*(CH_3_NO_2_) re‐dissolved in [D_3_]‐MeNO_2_ (Figure 2 a, b). (±)‐L1 was resolved into its constituent enantiomers by chiral HPLC,^11^ and each L1 enantiomer reacted with each of Λ‐[Ir(ppy)_2_(MeCN)_2_]⋅BF_4_ and Δ‐[Ir(ppy)_2_(MeCN)_2_]⋅BF_4_. As expected, the two combinations that were mis‐matched pairs of enantiomers gave poorly resolved ^1^H NMR spectra (Figures S10 and S11), whereas the two combinations that were matched pairs (presumably *M*‐Δ and *P*‐Λ) gave sharp spectra in short timeframes that were similar to the fully sorted cage mixture (Figures 2 d, S12, S13). ESI‐MS of matched and mis‐matched pairs are similar with all combinations showing cage formation (Figure S14). The observed ^1^H NMR spectral sharpening is therefore indicative of equilibration involving chiral self‐sorting of an initial mixture of cage stereoisomers; this was also seen in our previous studies of a [Pd_6_(L1)_8_]^12+^ cage but only the ligand was a chiral component.^12^ We could not resolve the sorted cages by analytical chiral HPLC.

**Figure 2:**
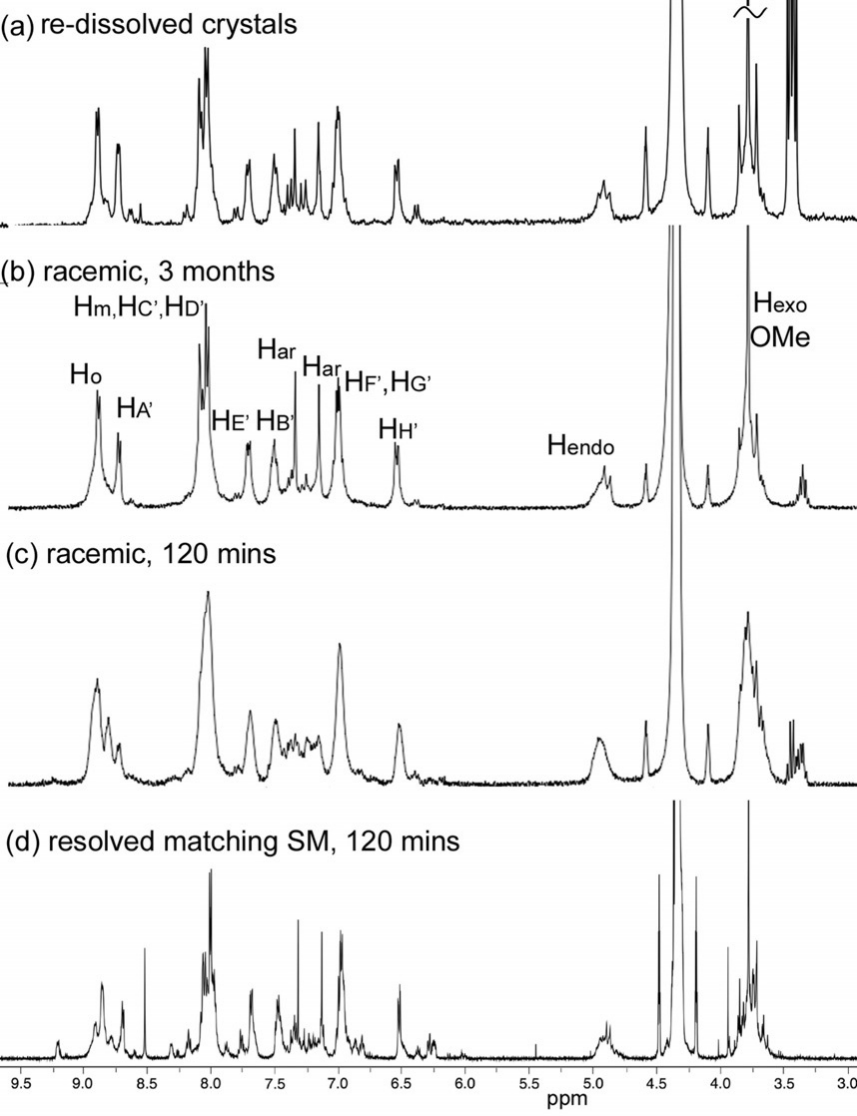
^1^H NMR spectra in CD_3_NO_2_ of (a) re‐dissolved racemic single crystals of *MM*‐ΛΛΛ and *PP*‐ΔΔΔ cages of **1**⋅3BF_4_; (b) (Λ,Δ)‐[Ir(ppy)_2_(MeCN)_2_]⋅PF_6_ and (±)‐L1 3 months after mixing; (c) (Λ,Δ)‐[Ir(ppy)_2_(MeCN)_2_]⋅PF_6_ and (±)‐L1 2 hrs after mixing; (d) matched pair of Δ‐[Ir(ppy)_2_(MeCN)_2_]^+^ and one L1 enantiomer after 2 hrs.

Homochiral metallo‐cages with tris‐chelate metal coordination are known both from achiral13a,13b and resolved chiral ligands.13c–13e Metallo‐cages that show homochiral self‐sorting from a racemic mixture of ligand enantiomers observed in solution are rare,^14^ although these include Pd^II^ metallo‐cryptophanes.8a The simultaneous chiral self‐sorting of both ligand and pre‐formed inert metallo‐tecton as reported here have not been previously reported. In a preliminary investigation of the influence of chiral guests on the self‐assembly of cage **1**, globular additives were included in 3:2 mixtures of (Λ,Δ)‐[Ir(ppy)_2_(MeCN)_2_]⋅PF_6_ and (±)‐L1. Addition of chiral *R*‐camphor or *S*‐camphor led to noticeably faster sharpening of the ^1^H NMR spectra than in their absence, but this was not observed for the addition of achiral adamantane (Figures S15–S20 in the Supporting Information). Interestingly, addition of the related anionic species *R‐*(or *S*‐)‐10‐camphorsulfonic acid to the reaction mixture prevents cage formation presumably as carboxylate is a competing ligand for the iridium (Figures S21 and S22).

The cages do not show self‐recognition of l‐ligand species. ESI‐MS of a MeNO_2_ solution of L1, L2 and [Ir(ppy)_2_(MeCN)_2_]⋅BF_4_ shows a statistical mixture of **1**:[{Ir(ppy)_2_}_3_(L1)(L2)]^3+^:**2** cage species (Figure 3). Mixing **1**⋅3BF_4_ and **2**⋅3BF_4_ in MeNO_2_ results in very slow exchange between L1 and L2 with appreciable ligand exchange only observed after four weeks, and near‐statistical mixing reached after ten weeks (Figure S6 in the Supporting Information). Thus, these cages have a high degree of kinetic stability but are not completely inert. It is interesting to note that this speciation behaviour is in contrast with recently reported [Pd_3_L_2_]^6+^ metallo‐cryptophanes, which exclusively formed homocages from two different l‐type ligands, with no ligand exchange.8a

**Figure 3:**
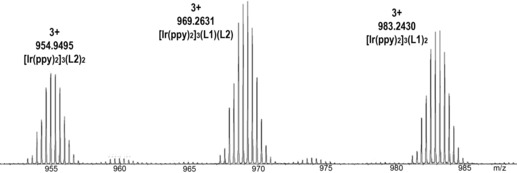
ESI‐MS of a 1:1:3 mixture of L1:L2: [Ir(ppy)_2_(MeCN)_2_]⋅BF_4_ in MeNO_2_ showing formation of a statistical mixture of homoleptic and heteroleptic cages.

The absorption spectra of **1** and **2** in dichloromethane (DCM) are similar to other [Ir(ppy)_2_($\hat{NN}$ )]^+^ systems,^7^ and characterised by two intense ligand centred (^1^LC) transitions between 260 and 320 nm localised on the ppy and three lower intensity broad bands below 380 nm that consist of spin‐allowed and spin‐forbidden mixed metal‐to‐ligand and ligand‐to‐ligand charge transfer (^1^MLCT/^1^LLCT and ^3^MLCT/^3^LLCT, respectively) transitions (Figure S26 in the Supporting Information). The weak CT transition observed for **1** at 470 nm was not reported for the monomeric [Ir(ppy)_2_(4‐pyCO_2_Et)_2_]^+^ (4‐pyCO_2_Et=4‐ethyl isonicotinate),10c suggesting increased conjugation in **1** due to the CTV scaffold. For both **1** and **2**, the excitation spectra in DCM match the absorption spectra and indicate a single photophysically active species.

Cages **1** and **2** are emissive in DCM solution and in the solid state. Upon photoexcitation of **1**, a broad and unstructured emission is observed both in DCM and in the powder (Figure 4 a) due to emission from a mixed ^3^MLCT/^3^LLCT state.^7^ The photoluminescence spectrum in the powder is red‐shifted (*λ*_max_=648 nm) compared to that in DCM (*λ*_max_=604 nm); however, **1** possesses similarly low *Φ*_PL_ of around 1 % and bi‐exponential decay kinetics in both media (Table 1). Due to the increased conjugation into the CTV scaffold, cage **1** shows red‐shifted emission and similar *Φ*_PL_ compared to [Ir(ppy)_2_(4‐pyCO_2_Et)_2_]^+^ (*λ*_max_=560 nm; *Φ*_PL_=2 %).10c Lusby's [{Ir(ppy)_2_}_6_(tcb)_4_]^6+^ cage also showed a red‐shifted emission (*λ*_max_=575 nm) when compared with the corresponding [Ir(ppy)_2_(NCPh)_2_]OTf complex (*λ*_max_=525 nm); however, unlike for cage **1** and other Ir(ppy)_2_ discrete supramolecular systems,^15^ the *Φ*_PL_ for the Lusby cage was enhanced compared with that of the mononuclear complex (*Φ*_PL_=4 % vs. *Φ*_PL_=<1 %).^4^

**Figure 4:**
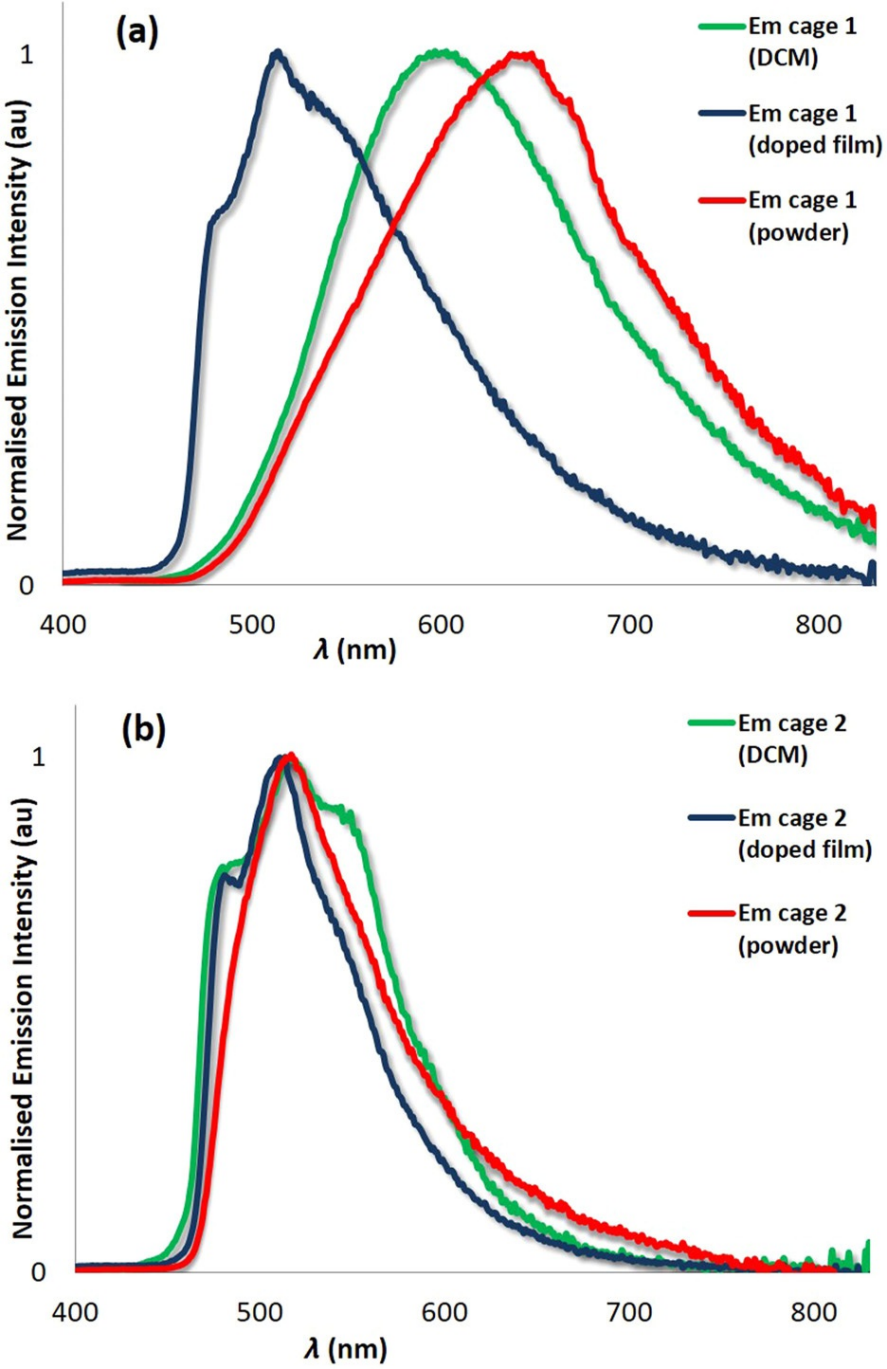
Normalised photoluminescence spectra of a) **1**⋅3BF_4_ and b) **2**⋅3BF_4_. Green lines are de‐aerated DCM solutions; blue lines are PMMA‐doped films with 5 wt % of cages spin‐coated on a quartz substrate; red lines are bulk powders.

**Table 1:** Photophysical properties of complexes **1**⋅3(BF_4_) and **2**⋅3(BF_4_).

Entry	*λ*_em_ (nm)			*Φ*_PL_(%)^[d]^			*τ*_e_ (ns)^[g]^		
	DCM^[a,b,f]^	Film^[c,f]^	Powder	DCM^[a]^	Film^[c,e]^	Powder^[e]^	DCM^[a]^	Film^[c]^	Powder
1	604	481 (0.7)	648	1	5.5	1.3	59 (0.7)	634 (0.4)	55 (0.6)
		514 (1)					129 (0.3)	2319 (0.6)	203 (0.4)
		556 (0.8)							
2	485 (0.8)	486 (0.8)	519	15	10	1.6	523 (0.4)	688 (0.7)	141 (0.4)
	516 (1)	515 (1)					887 (0.6)	3042 (0.3)	1175 (0.6)
	547 (0.6)	545 (0.6)							

[a] Measurements in degassed DCM at 298 K. [b] Quinine sulfate employed as the external reference (*Φ*_PL_=54.6 % in 0.5 m H_2_SO_4_ at 298 K). [c] PMMA‐doped films (5 wt % of cage) formed by spin‐coating deposition on a quartz substrate. [d] *Φ*_PL_ measurements were carried out under nitrogen (*λ*_exc_=360 nm). [e] Values obtained using an integrating sphere. [f] Principal emission peaks listed with values in parentheses indicating relative intensity. [g] *λ*_exc_=378 nm; values in parentheses are pre‐exponential weighting factors, in relative % intensity, of the emission decay kinetics.

To mitigate non‐radiative vibrational motion in the cage, we spin‐coated 5 wt % of **1** in polymethyl methacrylate (PMMA), which serves as an inert matrix. The emission in the thin film was blue‐shifted and more structured (*λ*_max_=514 nm) compared to both the powder and solution spectra. The *Φ*_PL_ of 5.5 % was enhanced as a result of the rigidity conferred by the PMMA host and the emission lifetimes were significantly longer (*τ*_e_=634 and 2319 ns).

The photoluminescence spectrum of cage **2** in DCM is more structured and blue‐shifted (*λ*_max_=516 nm) compared to **1**, indicating an emission that is more predominantly ligand‐centred (^3^LC; Figure 4 b). The blue‐shifted emission of **2** compared to **1** was expected considering the presence of the electron‐withdrawing ester moieties located on L1 in **1**, which stabilise the LUMO.10c Cage **2** shows a significantly enhanced *Φ*_PL_ and longer *τ*_e_ compared to **1** in DCM (*Φ*_PL_=15 %, *τ*_e_=523, 887 ns).

Unlike **1**, the emission of **2** as a powder is not significantly red‐shifted (*λ*_max_=519 nm), though the emission profile is less structured, showing less well‐resolved resolved vibrational bands as shoulders of the main emission peak. The emission profile for **2** in the PMMA‐doped thin film is likewise very similar to that in DCM. Although *Φ*_PL_ values are low in the powder (*Φ*_PL_=1.6 %), in the doped film they are higher (*Φ*_PL_=10 %). Emission lifetimes are expectedly longer in dopedfilms than in powder (Table 1). Attempts to synthesize an analogous mononuclear complex of 4‐phenoxymethylpyridine for comparison were not successful due to ligand oligomerization.

In summary, phosphorescent [{Ir(ppy)_2_}_3_(L)_2_]^3+^ metallo‐cryptophanes can be synthesized in high yields, with the CTV‐type ligands being able to accommodate torsion angles typical of [Ir(ppy)_2_(L)_2_] complexes to form rare examples of 3D Ir^III^ cyclometallated coordination cages. These cages undergo ligand exchange processes over months and show a remarkably high degree of homochiral self‐sorting of both ligand and metallo‐tecton, but not self‐recognition between similar l‐type ligands. Chiral sorting is enhanced by the presence of neutral chiral additives. For cage **1**, chiral self‐sorting occurs relatively rapidly upon crystallisation through an induced seeding effect, but on a timescale of months in solution. Luminescence properties of the two cages are quite distinct, pointing to an ability to tune the photophysical properties of these systems. Cage **2** showed an enhanced and blue‐shifted emission compared to **1**, reaching a *Φ*_PL_ of 15 % in DCM solution and 10 % in doped film. These are promising systems for a variety of applications including semiochemical hosts, photoredox catalysts and in energy conversion materials.

## Conflict of interest

The authors declare no conflict of interest.

## Supporting information

As a service to our authors and readers, this journal provides supporting information supplied by the authors. Such materials are peer reviewed and may be re‐organized for online delivery, but are not copy‐edited or typeset. Technical support issues arising from supporting information (other than missing files) should be addressed to the authors.

Supplementary
