# Supplementary material for: Homochiral Self‐Sorted and Emissive IrIII Metallo‐Cryptophanes
Source: Chemistry. 2017 Apr 20;23(26):6290–4. doi: 10.1002/chem.201701348 (PMC5499720; doi:10.1002/chem.201701348)
Supplement: Supplementary file 1 — Supplementary [file CHEM-23-6290-s001.pdf]

CHEMISTRY
A European Journal

Supporting Information

Homochiral Self-Sorted and Emissive Ir^{III} Metallo-Cryptophanes

Victoria E. Pritchard,[a] Diego Rota Martir,[b] Samuel Oldknow,[a] Shumpei Kai,[c] Shuichi Hiraoka,[c] Nikki J. Cookson,[a] Eli Zysman-Colman,*[b] and Michaele J. Hardie*[a]

chem_201701348_sm_miscellaneous_information.pdf

Homochiral self-sorted and emissive Ir(III) metallo-cryptophanes

Victoria E. Pritchard,[a] Diego Rota Martir,[b] Samuel Oldknow,[a] Shumpei Kai,[c] Shuichi Hiraoka,[c] Nikki J. Cookson,[a] Eli Zysman-Colman*,[b] and Michaele J. Hardie*[a]

[a] School of Chemistry, University of Leeds, Woodhouse Lane, Leeds, LS2 9JT, UK

[b] Organic Semiconductor Centre, EaStCHEM School of Chemistry, University of St Andrews, St Andrews, Fife, KY16 9ST, UK.

[c] Department of Basic Science, Graduate School of Arts and Sciences, The University of Tokyo, 3-8-1 Komaba, Meguro-ku, Tokyo 153-8902, Japan

Supplementary Material

1. Synthesis

2. Mass spectrometry

3. NMR studies of cage assembly

4. MS and NMR of Cage Assembly in the presence of guests

5. X-Ray Crystallography

6. Photophysical studies

1. Synthesis

Tris(isonicotinoyl)-cyclotriguaiacylene (**L1**),[1] *tris*(4-pyridyl-methyl)-cyclotriguaiacylene (**L2**) [2] and $[Ir(ppy)_2(MeCN)_2]\cdot BF_4$ [3] where ppy = 2-phenylpyridinato were synthesized according to literature methods, with resolved intermediate $[Ir(ppy)_2(Cl)_2]_2$ and enantiopure solvento-complexes Λ -$[Ir(ppy)_2(MeCN)_2]\cdot BF_4$ and Δ-$[Ir(ppy)_2(MeCN)_2]\cdot BF_4$ were obtained by previously reported protocols.[4] All other chemicals were obtained from commercial sources and were used without further purification. NMR spectra were recorded by automated procedures on a Bruker DPX 300 MHz NMR spectrometer, a Bruker Avance 500 MHz NMR spectrometer or a Jeol 600ii 600 MHz NMR spectrometer at room temperature. DOSY NMR experiments were performed using a Jeol ECA 600ii 600 MHz spectrometer. Data were recorded at 293 K using a 5mm probe. DOSY NMR experiments were performed using the bipolar pulse pair stimulated echo (BPPSTE) operating in the ONESHOT experiment. Additional parameters: number of different gradient levels, 20; gradient stabilisation delay, 0.002 s; gradient length, 0.005 s, diffusion delay, 0.1 s; relaxation delay, 10 s; Kappa (unbalancing factor), 0.2 s. DOSY data was processed using the DOSYtoolbox version 2.5, developed by Mathias Nilsson, University of Manchester.[5] Electrospray mass spectra (ES-MS) were measured on a Bruker Maxis Impact instrument in positive ion mode, or a Waters Xevo G2-S Tof mass spectrometer.

Infra-red spectra were recorded as solid phase samples on a Bruker ALPHA Platinum ATR. Elemental analyses were performed on material that had been washed with diethyl ether, subsequently dried under vacuum and then exposed to the atmosphere.

The two enantiomers of **L1** were separated by chiral HPLC (column: CHIRAL OD-H, eluent: MeCN and MeOH (4:1 (v/v))), it is uncertain which isomer is *P* or *M* and thus the isomers are indicated by **F2** and **F4** according to their fraction number (Figure S1). ^{1}H NMR of **F2** and **F4** in CD_3NO_2 show additional peaks which may be due to minor decomposition or intermediate conformers from racemisation via crown-saddle-crown.

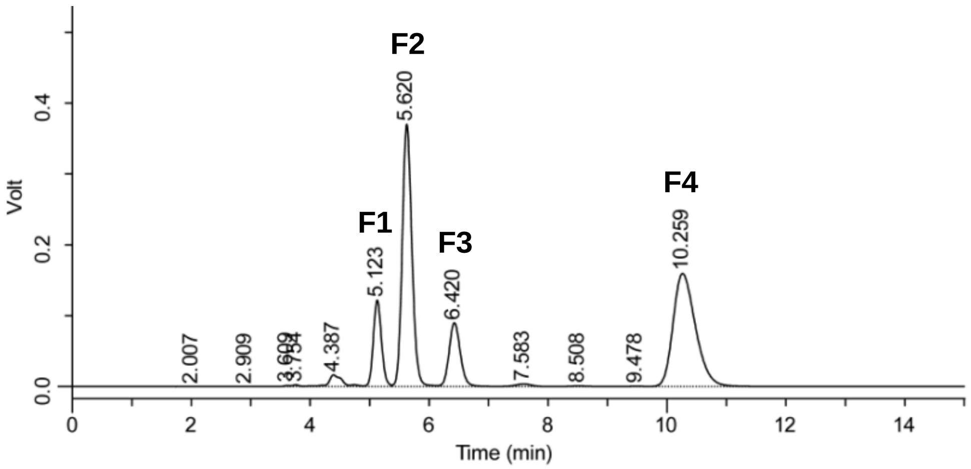

Figure S1. *Chromatogram of chiral resolution of the tritopic ligand L by chiral HPLC.* ***F2*** *and* ***F4*** *are the enantiomers of the ligand.*

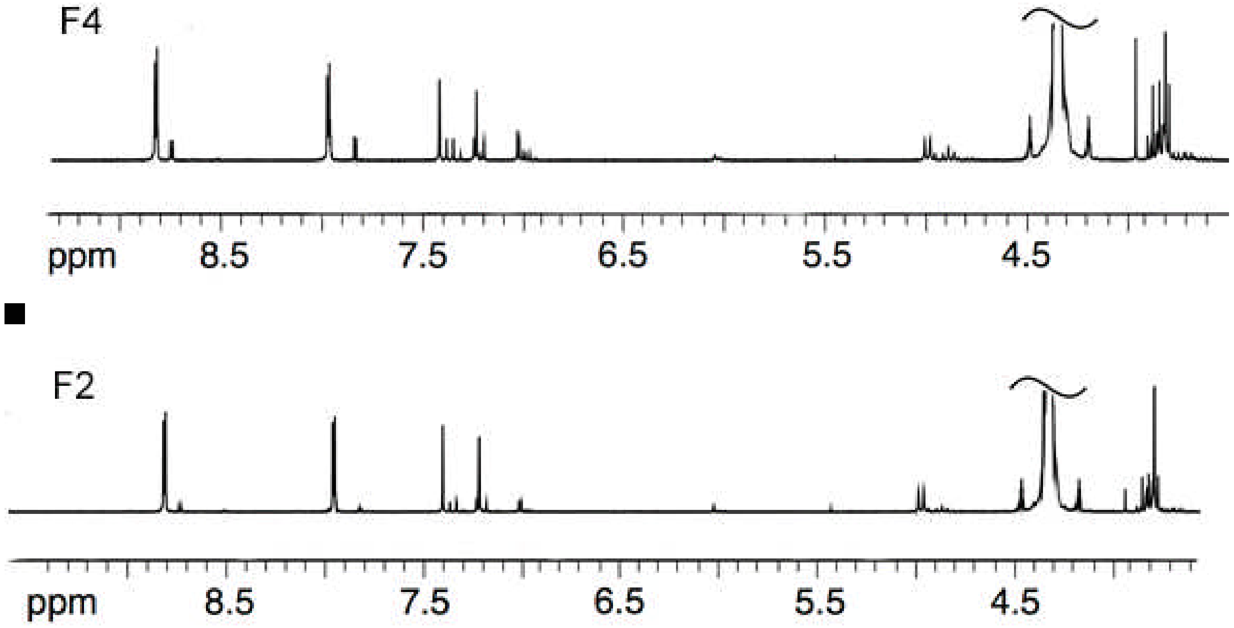

Figure S2. *^{1}H NMR (500 MHz, CD_3NO_2, 298 K) of* ***L1*** *enantiomers.*

$\{[Ir(ppy)_2]_3(L1)_2\}\cdot 3(BF_4)$ and $\{[Ir(ppy)_2]_3(L1)_2\}\cdot 3(PF_6)$, cage 1

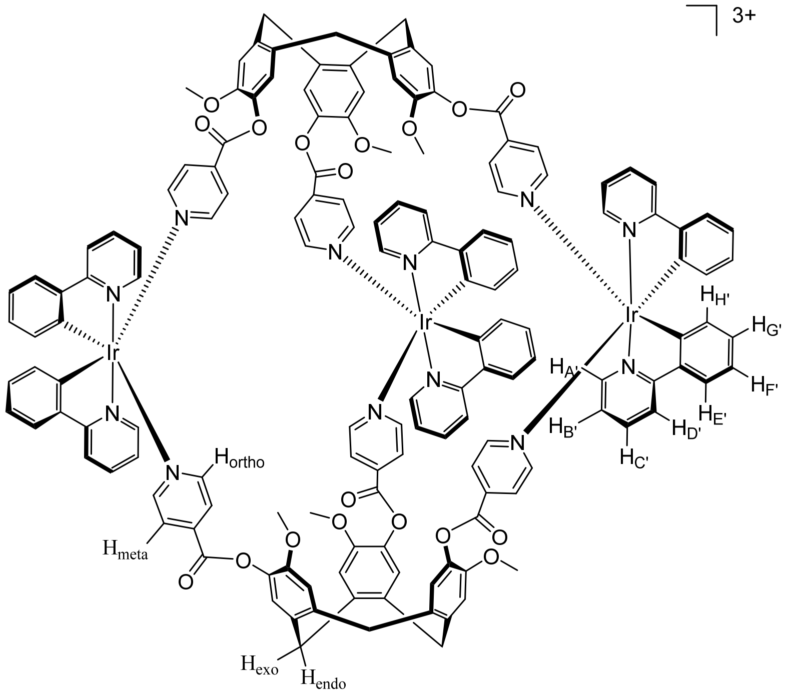

$[Ir(ppy)_2(MeCN)_2]\cdot BF_4$ (0.036 g, 0.054 mmol) and (±)-**L1** (0.025 g, 0.035 mmol) were combined in nitromethane solvent (5 mL) and stirred for 12 hours at room temperature. The solution was concentrated *in vacuo* and diethyl ether was added to the solution to give $\{[Ir(ppy)_2]_3(L1)_2\}\cdot 3(BF_4)$ as a bright yellow powder (0.050 g, 90%). FT-IR, solid state (cm^{-1}); 1748 (s, C=O), 1608, 1507, 1479, 1418, 1269, 1056 (s, BF_4 anion), 758, 698.

$[Ir(ppy)_2(MeCN)_2]\cdot PF_6$ (0.037 g, 0.052 mmol) and (±)-**L1** (0.025 g, 0.035 mmol) were combined in nitromethane solvent (5 mL) and stirred for 12 hours at room temperature. The solution was concentrated *in vacuo* and diethyl ether was added to the solution to give $\{[Ir(ppy)_2]_3(L1)_2\}\cdot 3(PF_6)$ as a bright yellow powder (0.051 g, 88%).

Consistent MS were obtained for both salts (see Figure S1). TOF-MS ESI: *m/z* = 983.1120 $\{[Ir(ppy)_2]_3(L1)_2\}^{3+}$, 862.3934 $\{[Ir(ppy)_2]_2(L1)\}^{2+}$, 1224.5712 $\{[Ir(ppy)_2]_2(L1)_2\}^{2+}$.

Micro-analysis for $C_{150}H_{114}B_3F_{12}Ir_3N_{12}O_{18}$ (Calculated, Found); C (56.13, 52.23), H (3.58, 3.77), N (5.24, 5.65)

^{1}H NMR studies were carried out in d_3-$MeNO_2$. **L1** was suspended in deuterated $MeNO_2$ in an NMR tube. The tube was sonicated for ten minutes and heated (heat gun) until all the material dissolved. $[Ir(ppy)_2(MeCN)_2]\cdot PF_6$ was dissolved in deuterated nitromethane, and the individual spectrum recorded for comparison. The two solutions were mixed together and an immediate colour change was observed, from the green of the iridium metallotecton solution to bright yellow. Immediate broadening of the resultant spectra was observed, indicating coordination and formation of a larger species. ^{1}H NMR (300 MHz, CD_3NO_2) δ 9.12 – 8.54 (bm, 3H, $H_{A'}$/H_{ortho}), 8.05 (bd, J = 15.5 Hz, 4H, $H_{C'}$/$H_{D'}$/H_{meta}), 7.88 – 7.67 (bm, 1H, $H_{E'}$), 7.62 – 7.10 (bm, 3H, $H_{B'}$/2xH_{aryl}), 7.09 – 6.72 (bm, 2H, $H_{F'}$/$H_{G'}$), 6.54 (bs, 1H, $H_{H'}$), 4.97 (d, J = 15.3 Hz, 1H, H_{endo}), 3.80 (bd, J = 14.4, 10.2 Hz, 4H, H_{exo}/OMe).

X-ray quality crystals were grown by vapour diffusion of diethyl ether into a nitromethane solution of $\{[Ir(ppy)_2]_3(L1)_2\}\cdot 3(BF_4)$.

Cages are not stable in coordinating solvents, decomposing rapidly in DMSO and more slowly in MeCN.

{[Ir(ppy)$_2$]$_3$(L2)$_2$}·3(BF$_4$) and {[Ir(ppy)$_2$]$_3$(L2)$_2$}·3(PF$_6$), cage 2

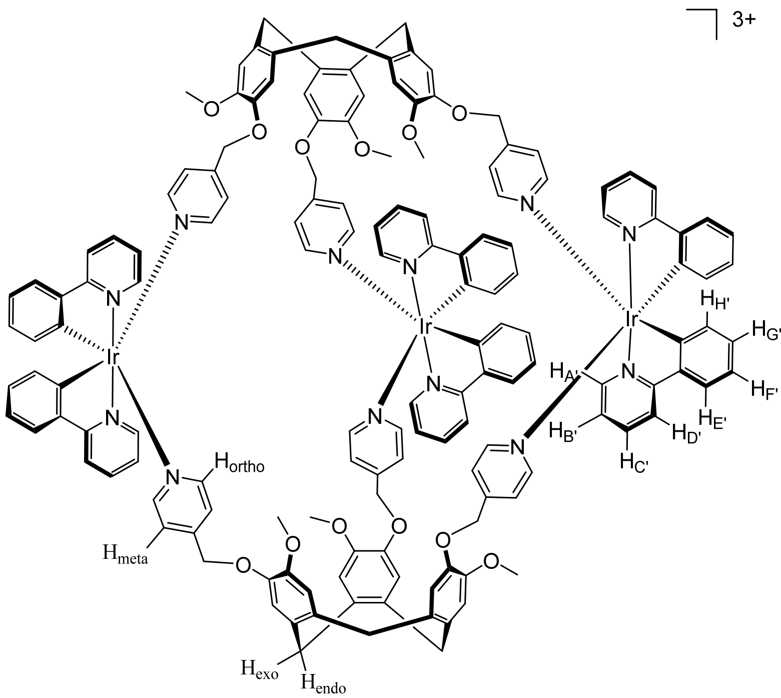

[Ir(ppy)$_2$(MeCN)$_2$]·BF$_4$ (0.036 g, 0.054 mmol) and **(±)-L2** (0.025 g, 0.037 mmol) were combined in nitromethane solvent (5 mL) and stirred for 12 hours at room temperature. The solution was concentrated *in vacuo* and diethyl ether was added to the solution to give {[Ir(ppy)$_2$]$_3$(**L2**)$_2$}·3(BF$_4$) as a bright yellow powder (0.056 g, 97%). TOF-MS ESI: *m/z* = 955.2853 {[Ir(ppy)$_2$]$_3$(L2)$_2$}$^{3+}$, 841.7365 {[Ir(ppy)$_2$]$_2$(L2)}$^{2+}$, 1182.3779 {[Ir(ppy)$_2$](L2)}$^{2+}$, FT-IR, solid state (cm^{-1}); 1608, 1508, 1479, 1267, 1059 (s, BF$_4$ anion), 758.

Micro-analysis for $C_{150}H_{126}B_3F_{12}Ir_3N_{12}O_{12}$ (calculated, found); C (57.64, 52.40), H (4.06, 4.08), N (5.38, 5.36)

^{1}H NMR studies were carried out in *d*$_3$-MeNO$_2$, [Ir(ppy)$_2$(MeCN)$_2$]·PF$_6$ and **L2** were dissolved in deuterated nitromethane, and the individual spectrum recorded for comparison. The two solutions were mixed together and an immediate colour change was observed, from the green of the iridium metallotecton solution to bright yellow. Immediate broadening of the resultant spectra was observed, indicating coordination and formation of a larger species. ^{1}H NMR (300 MHz, CD_3NO_2) δ 8.58 (bm, *J* = 27.4 Hz, 3H, H$_{A'}$/H$_{ortho}$), 8.04 (bm, 2H, H$_{C'}$/H$_{D'}$), 7.59 (bm, *J* = 48.9 Hz, 4H, H$_{E'}$/H$_{B'}$/H$_{meta}$), 7.05 (bm, *J* = 48.4 Hz, 4H, H$_{F'}$/H$_{G'}$/2xH$_{aryl}$), 6.50 (bs, 1H, H$_{H'}$), 5.39 – 4.89 (m, 2H, CH$_2$), 4.81 (bs, 1H, H$_{endo}$), 3.99 – 3.36 (bm, 4H, H$_{exo}$/OMe).

2. Mass Spectrometry

{[Ir(ppy)$_2$]$_3$(L1)$_2$}$^{3+}$, cage 1

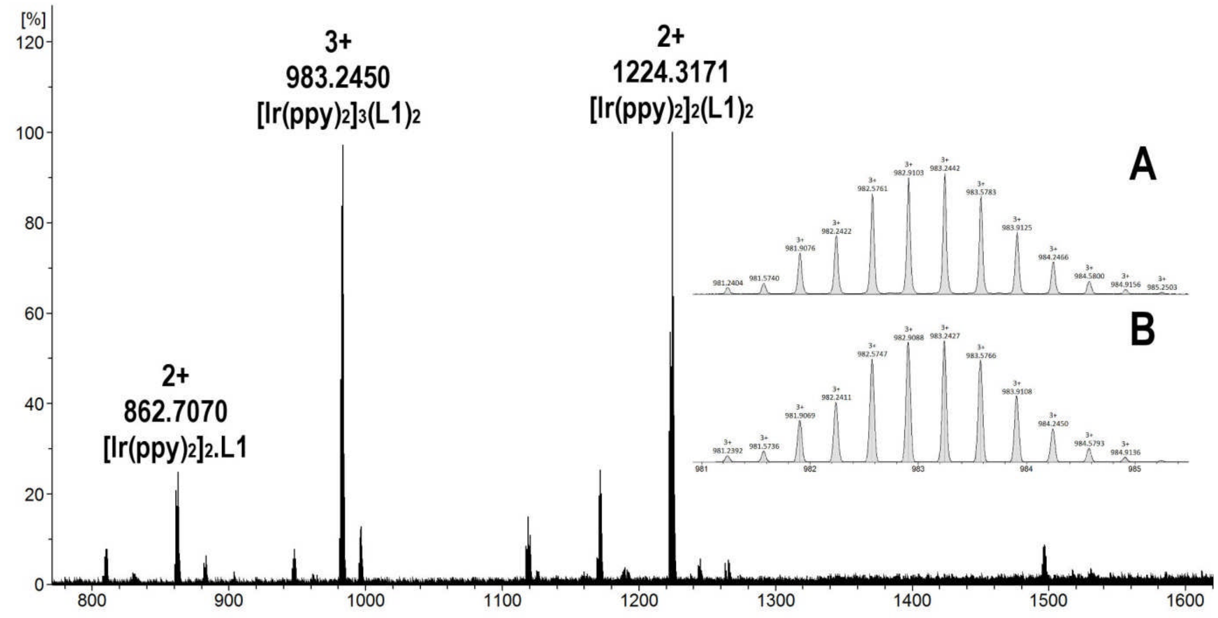

(a)

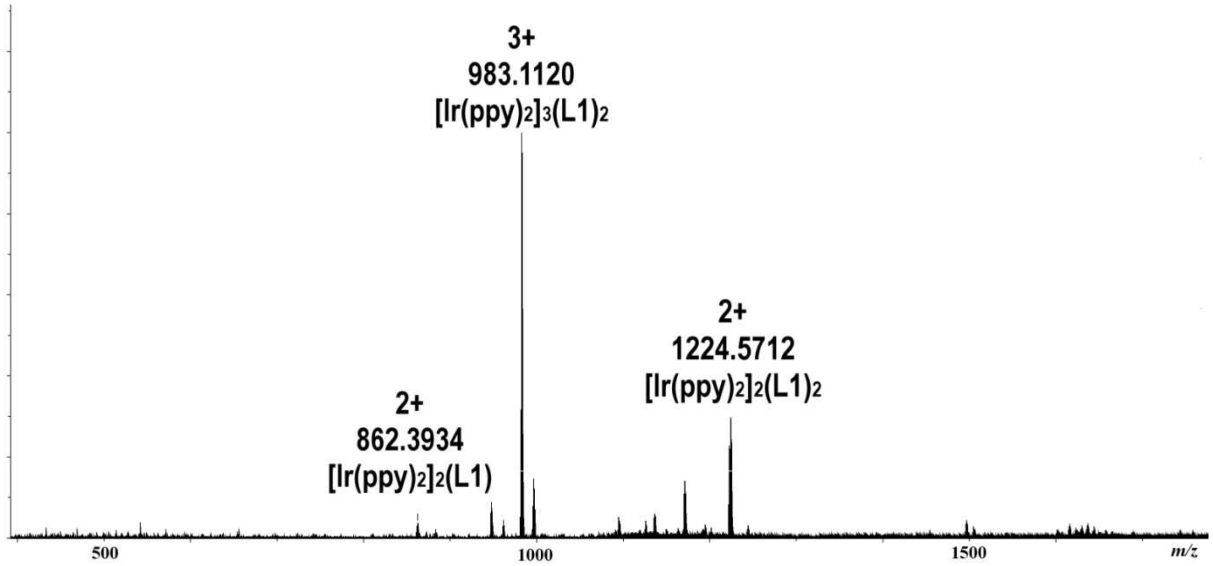

(b)

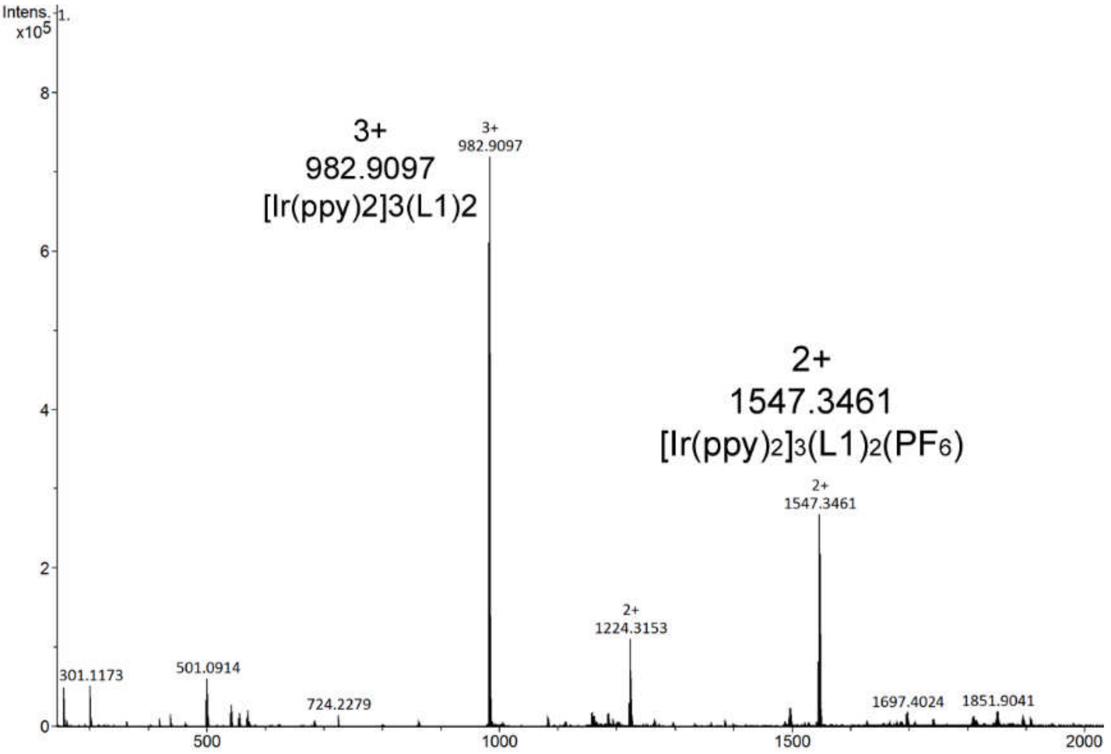

(c)

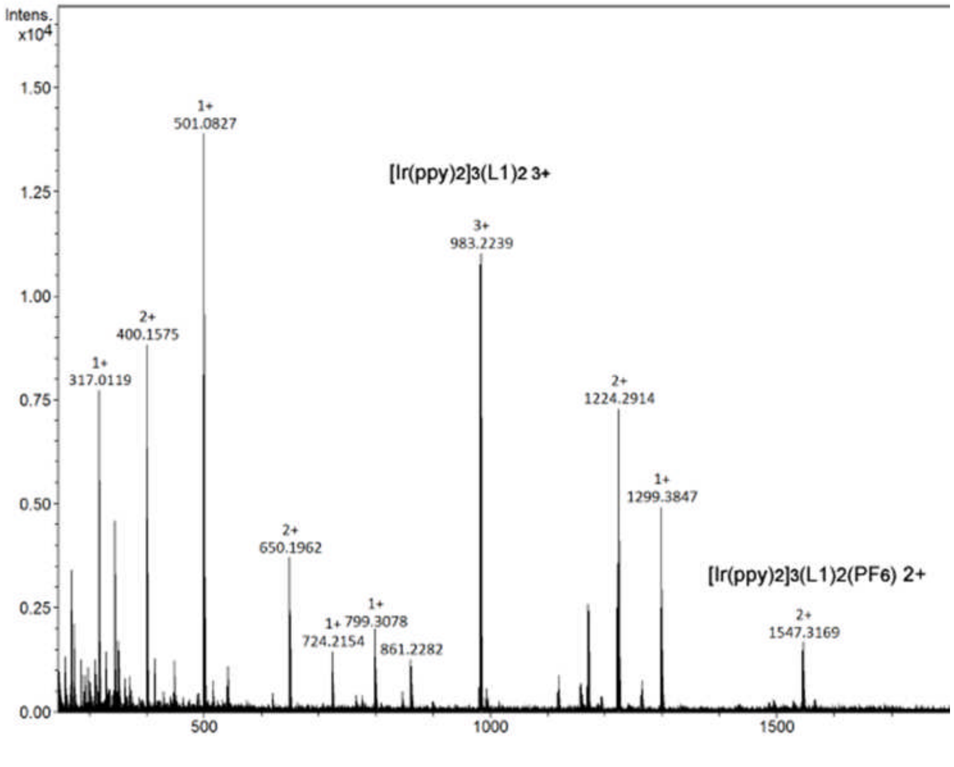

(d)

Figure S3: *High Resolution ESI-MS of cage* ***1****. (a)* $\{[Ir(ppy)_2]_3(\mathbf{L1})_2\}\cdot 3(BF_4)$ *collected after 12hrs in solution showing complex* $\mathbf{1}\cdot 3(BF_4)$ *along with fragmentation products,* M_3L_2 *peaks shown inset A: measured, B: calculated isotope pattern, where* $M=[Ir(ppy)_2]^+$*. (b)* $\{[Ir(ppy)_2]_3(\mathbf{L1})_2\}\cdot 3(PF_6)$ *collected after 12hrs in solution showing complex complex* $\mathbf{1}\cdot 3(PF_6)$ *along with fragmentation products; (c)* $\{[Ir(ppy)_2]_3(\mathbf{L1})_2\}\cdot 3(PF_6)$ *collected after 3 months in solution; (d) powder cage redissolved in* CH_2Cl_2*.*

{[Ir(ppy)$_2$]$_3$(L2)$_2$}$^{3+}$, cage 2

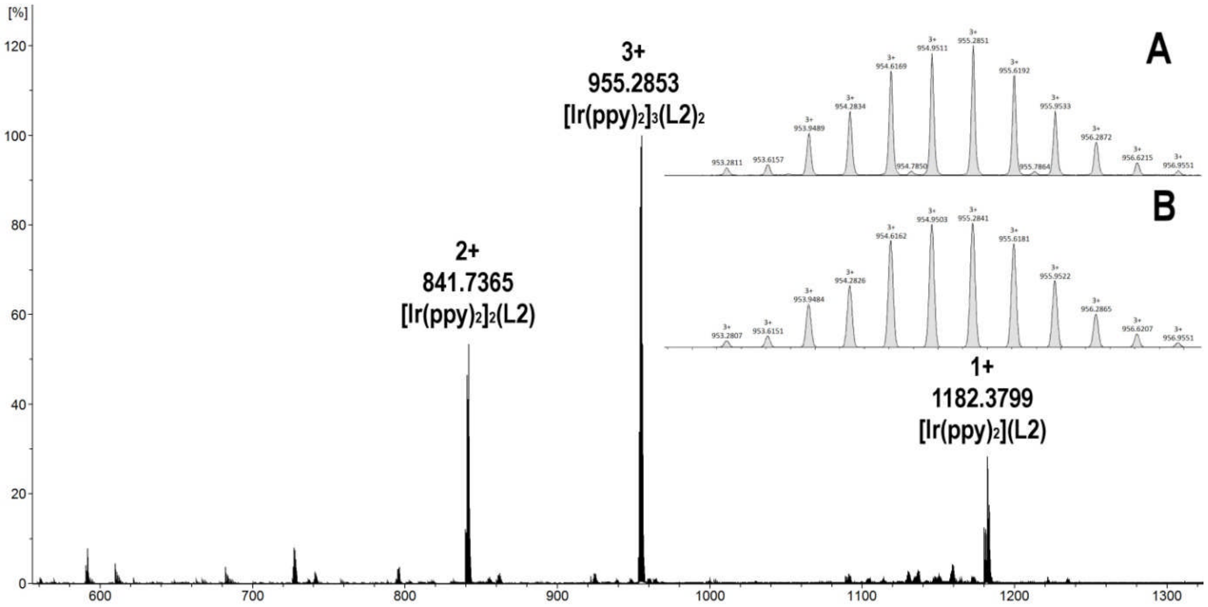

(a)

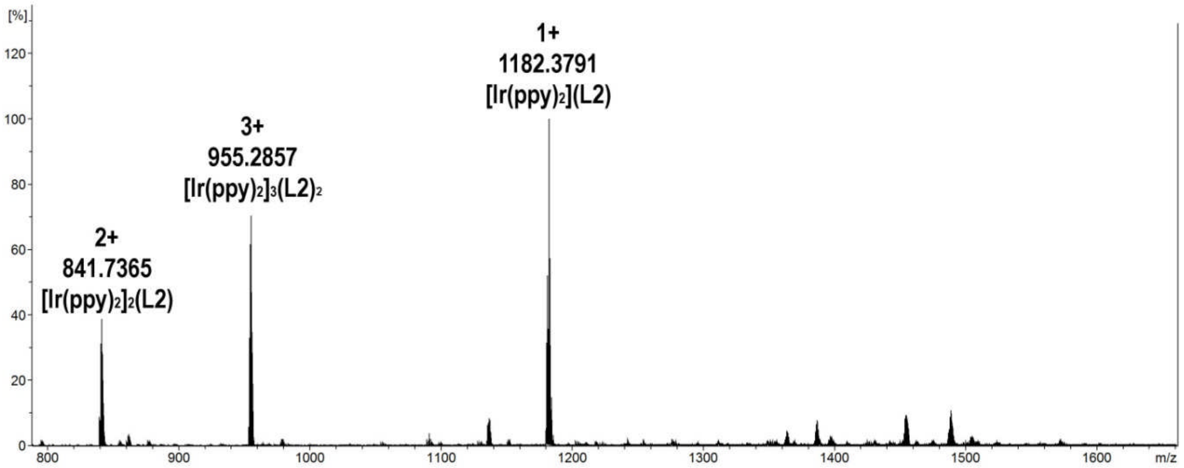

(b)

***Figure S4**: High Resolution ESI-MS after overnight in solution (a) {[Ir(ppy)$_2$]$_3$(**L2**)$_2$}·3(BF$_4$), complex **2**·3(BF$_4$) along with fragmentation products, M$_3$L$_2$ peaks shown inset a) measured b) calculated isotope pattern, where M=[Ir(ppy)$_2$]$^+$; (b) {[Ir(ppy)$_2$]$_3$(**L2**)$_2$}·3(PF$_6$), complex **2**·3(PF$_6$) along with fragmentation products.*

Heteroleptic metallocryptophanes

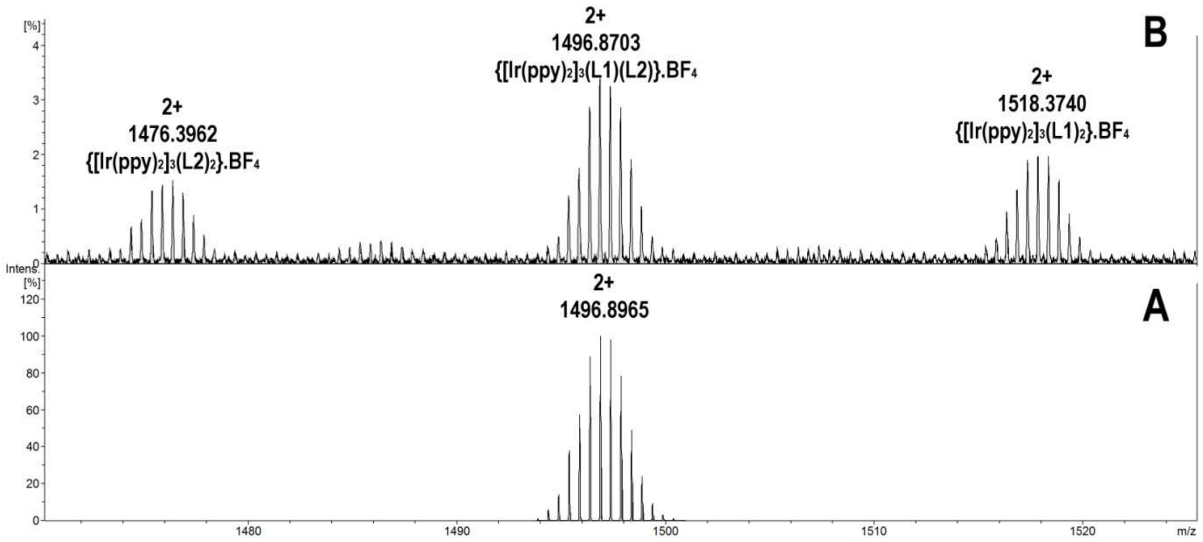

(i)

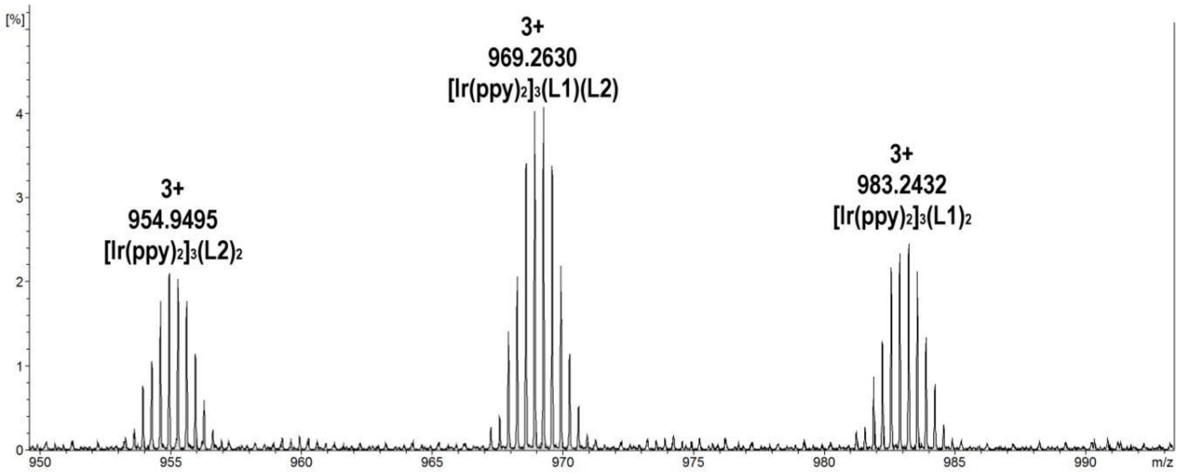

(ii)

***Figure S5**: Section of the high resolution ESI-MS of a mixture of three equivalents of $[Ir(ppy)_2(MeCN)_2]\cdot BF_4$ and one equivalent each of **L1** and **L2** in nitromethane (i) taken after 8 hrs of stirring at room temperature, and showing a statistical mixture of homoleptic and heteroleptic cages. M = $[Ir(ppy)_2]^+$ the calculated spectrum of the heteroleptic cage is shown below in A); (ii) after 4 months equilibration.*

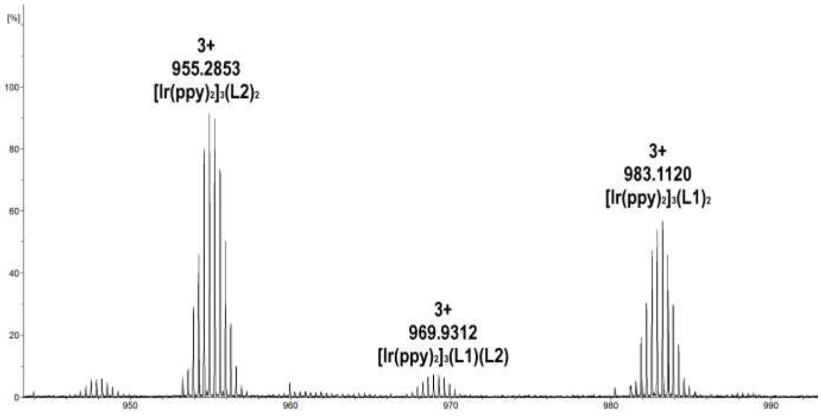

(a)

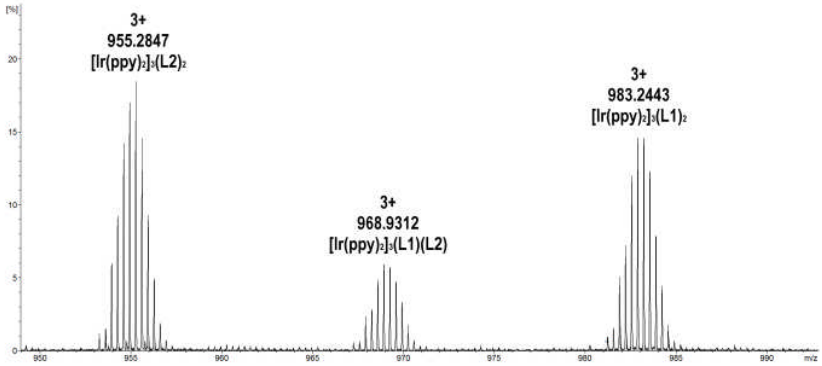

(b)

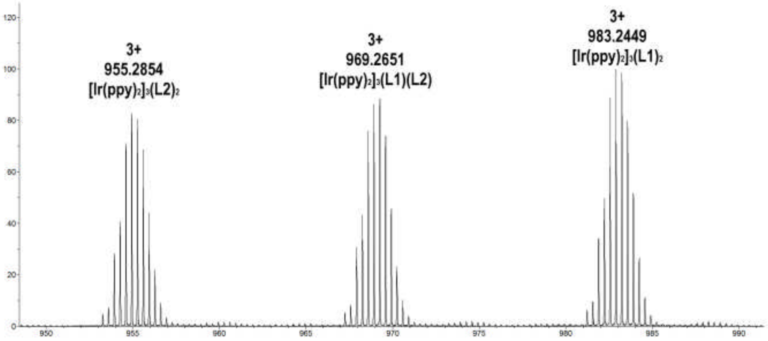

(c)

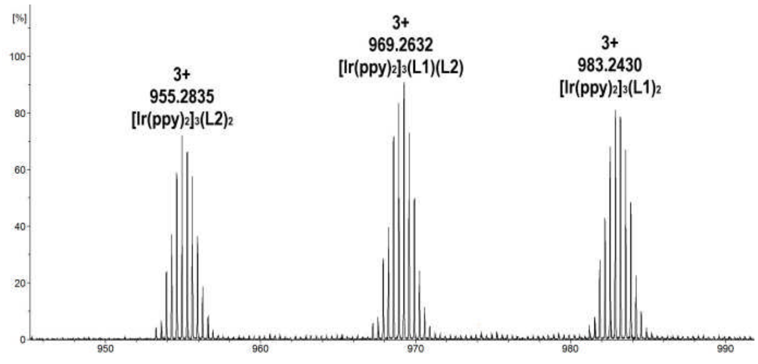

(d)

Figure S6: *High resolution ESI-MS of powdered* ***1***$\cdot 3BF_4$ *and* ***2***$\cdot 3BF_4$ *combined in* $MeNO_2$ *after a) 24hrs, b) 4 weeks, and c) 6 weeks, d) 10 weeks.*

3. NMR studies

{[Ir(ppy)$_2$]$_3$(L1)$_2$}$^{3+}$, cage 1

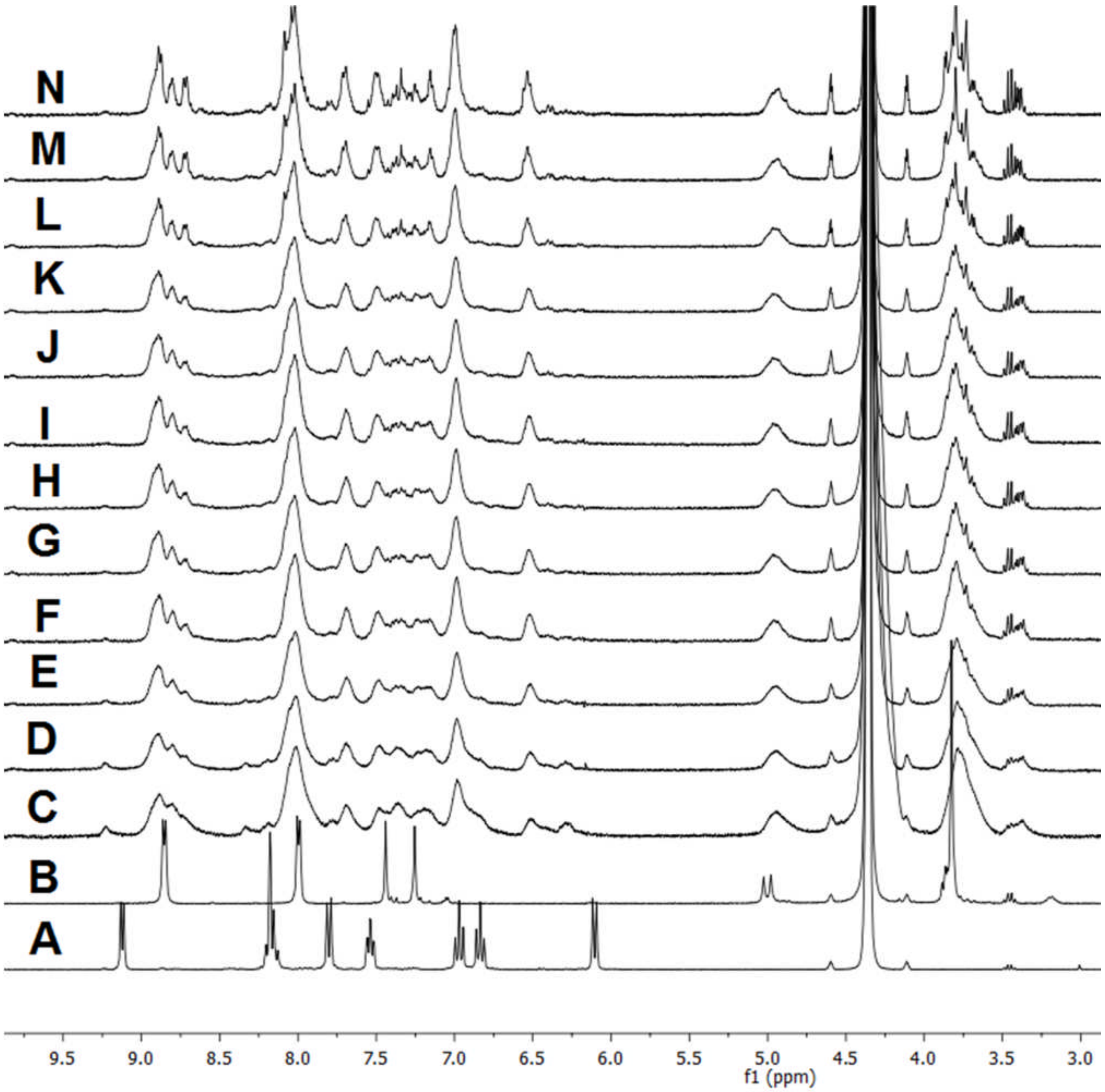

Figure S7: *^{1}H NMR (d_3-MeNO$_2$) of **1**·3PF$_6$ (a) following the initial formation of **1**·3PF$_6$ at room temperature. A = (Δ,Λ)-[Ir(ppy)$_2$(MeCN)$_2$]·PF$_6$; B= (±)-**L1**; C = initial: D = 15 mins; E = 30 mins; F = 45 mins; G = 60 mins; H = 75 mins; I = 90 mins; J = 105 mins; K = 120 mins; L = 18 hrs; M = 100 hrs; N = 124 hrs.*

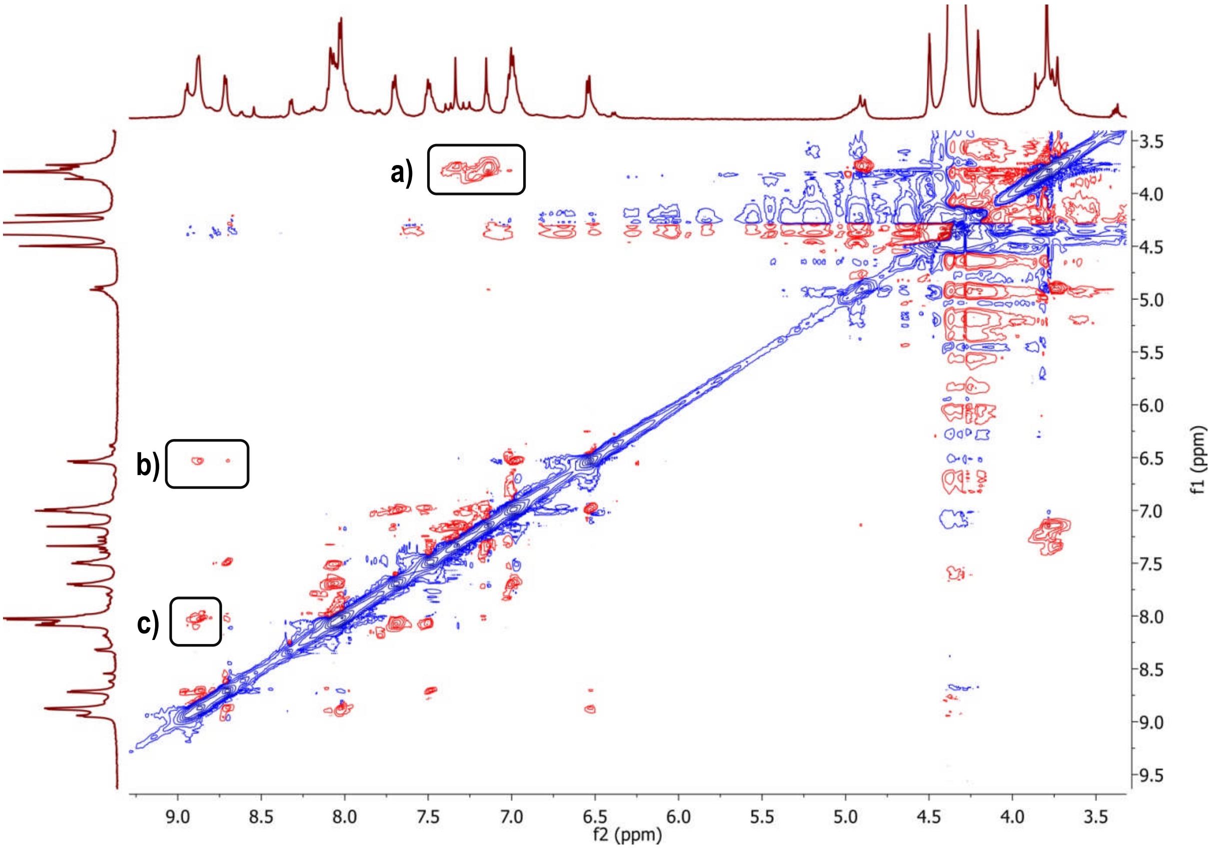

Figure S8: *ROESY spectrum of* ***1****·3PF$_6$ in d$_3$-MeNO$_2$ solvent, a) coupling between exo/methoxy protons and aryl-CTG protons, b) coupling between $H_{H'}$ on the phenylpyridine ancillary ligand and the ortho-pyridyl proton on* ***L1****, c) coupling between ortho/meta pyridyl protons and likely inter-ligand phenylpyridine coupling.*

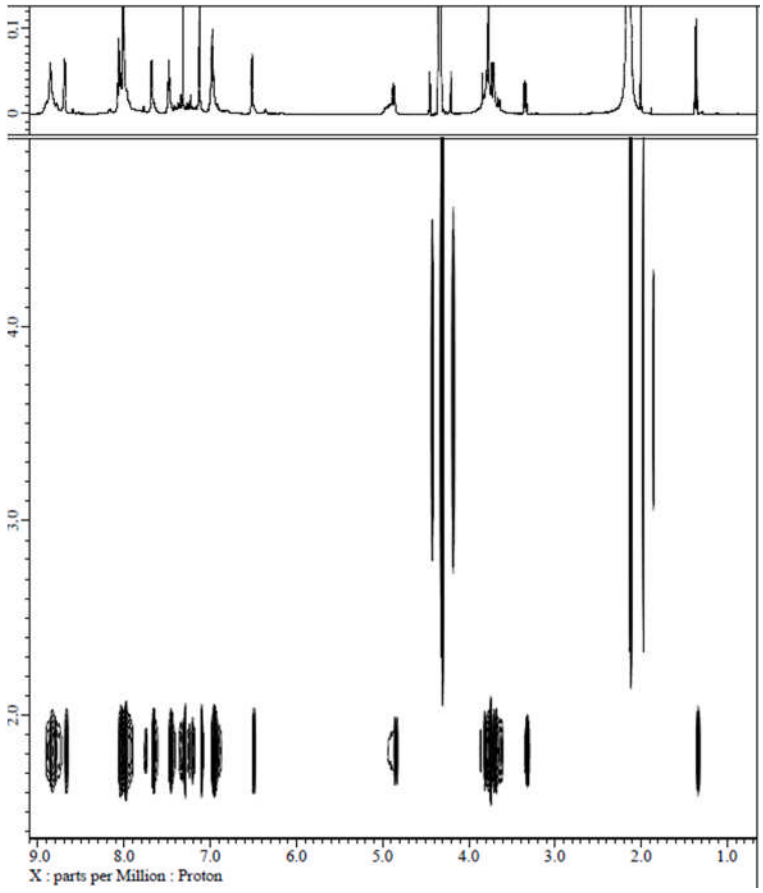

(a)

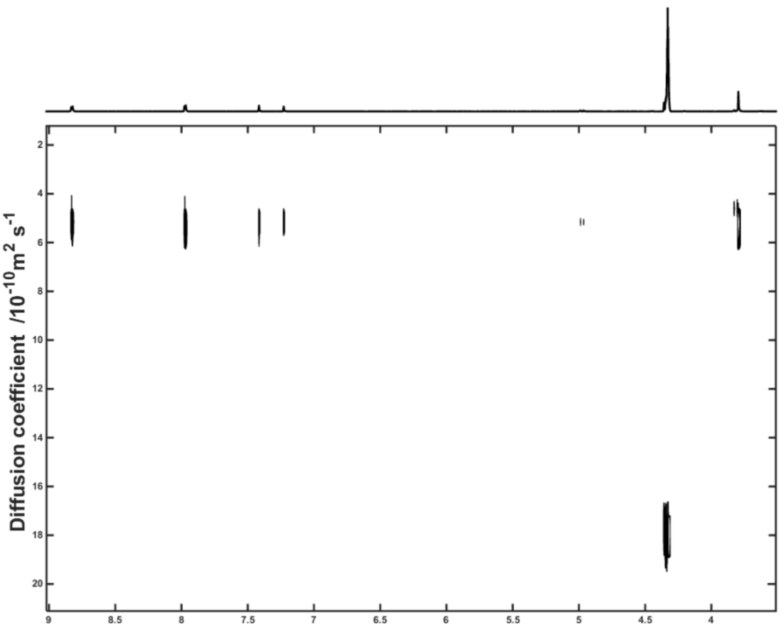

(b)

***Figure S9**:(a) DOSY NMR spectrum of equilibrated complex **1**·3PF$_6$ in d$_3$-MeNO$_2$, showing a D_c of 1.82x10^{-10} m^{2}s^{-1}; (b) DOSY NMR spectrum of **L1** in d$_3$-MeNO$_2$, showing a D_L of 5.2x10^{-10} m^{2}s^{-1}. Hydrodynamic radius can be estimated using Stokes-Einstein equation: $R_H = kT/6\pi\eta D$ where k = Boltzmann constant, T = 20K, η = solvent viscosity η = 0.620 x 10^{-3} Pa.s.*

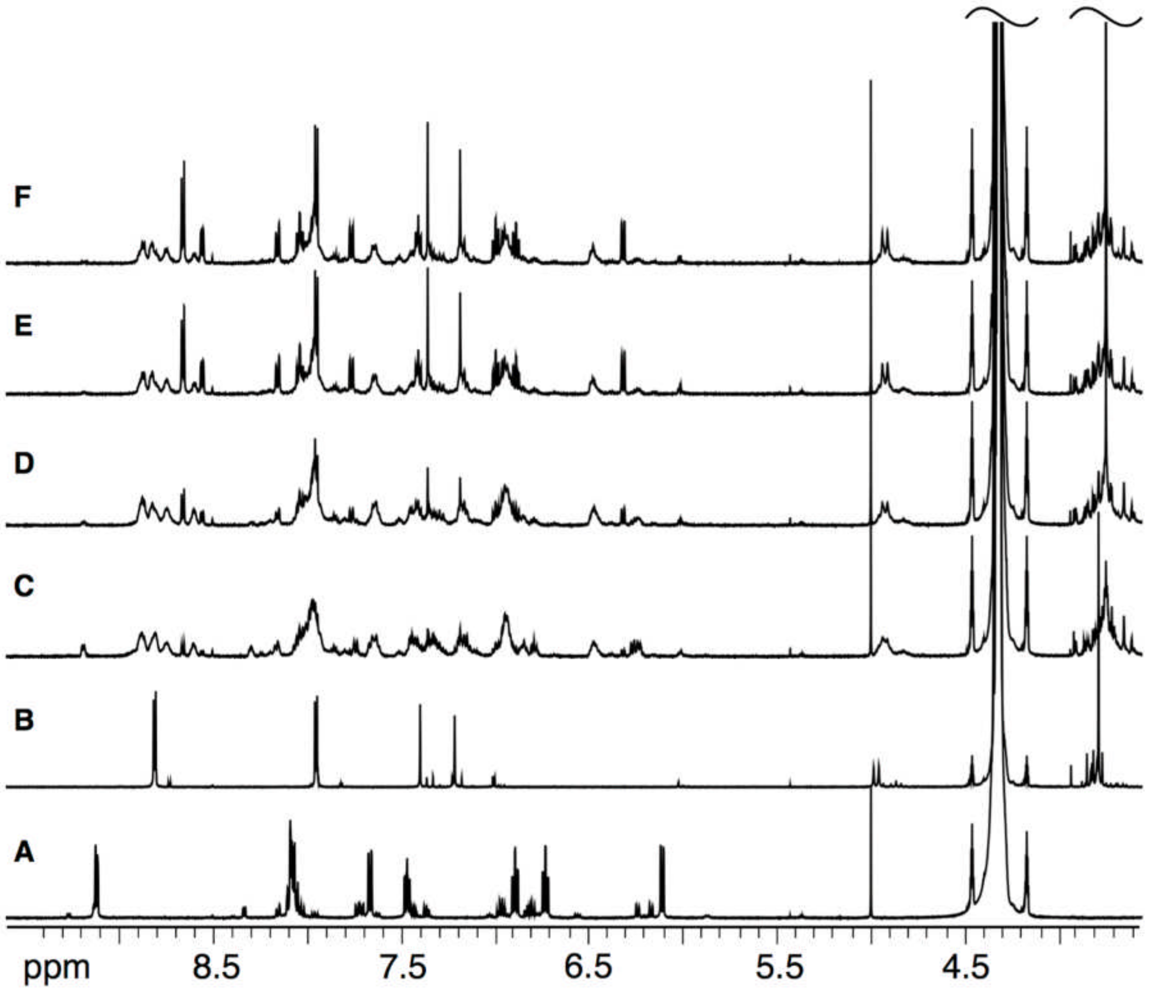

Figure S10. *^{1}H NMR spectra (500 MHz, CD_3NO_2, 298 K, [**F2**] = 2.2 mM) of the complexation of Λ-[Ir(ppy)$_2$(MeCN)$_2$]·BF_4 and one of the enantiomers of the tritopic ligand, **F2**. (A) Λ-[Ir(ppy)$_2$(MeCN)$_2$]·BF_4, (B) **F2**, (C) 10 min, (D) 2 h, (E) 2 days, (F) 24 days. Mis-matched pair of enantiomers.*

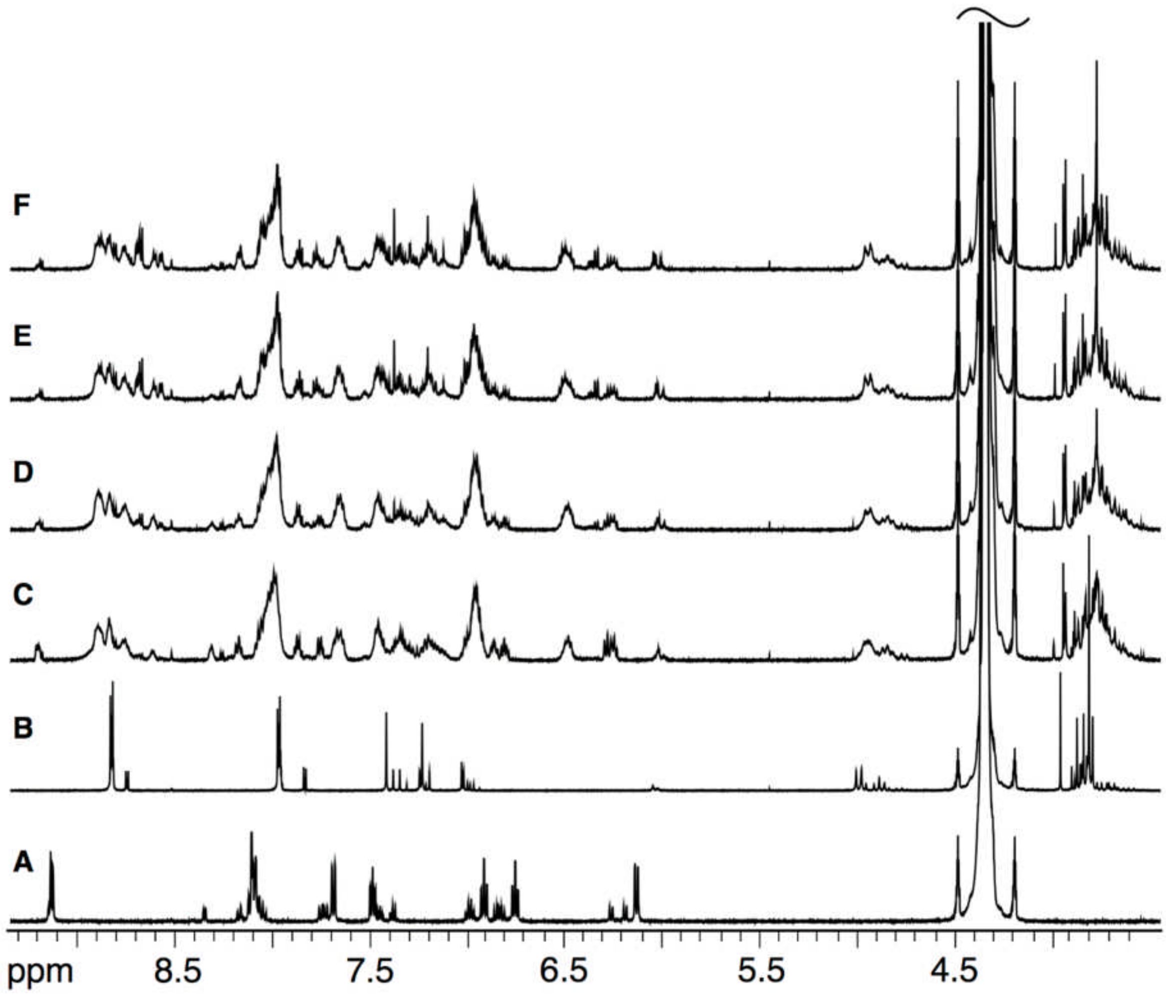

Figure S11. *^{1}H NMR spectra (500 MHz, CD_3NO_2, 298 K, [**F4**] = 2.2 mM) of the complexation of Δ-[Ir(ppy)$_2$(MeCN)$_2$]·BF_4 and one of the enantiomers of tritopic ligand (L), **F4**. (A) Δ-[Ir(ppy)$_2$(MeCN)$_2$]·BF_4, (B) **F4**, (C) 10 min, (D) 2 h, (E) 2 days, (F) 19 days. Mis-matched pair.*

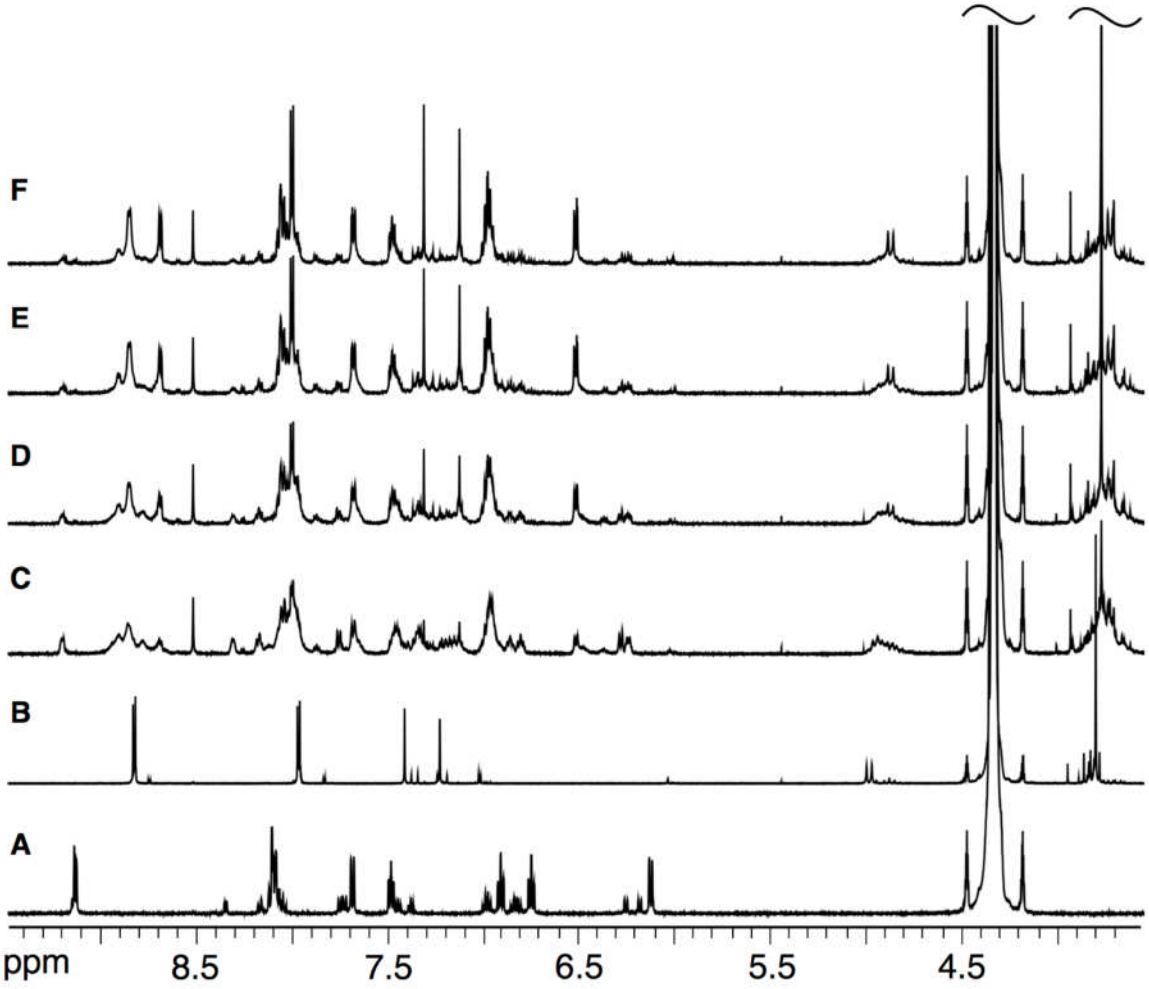

Figure S12. *^{1}H NMR spectra (500 MHz, CD_3NO_2, 298 K, [**F2**] = 2.2 mM) of the complexation of Δ-[Ir(ppy)$_2$(MeCN)$_2$]·BF_4 and one of the enantiomers of tritopic ligand (L), **F2**. (A) Δ-[Ir(ppy)$_2$(MeCN)$_2$]·BF_4, (B) **F2**, (C) 10 min, (D) 2 h, (E) 2 days, (F) 14 days. Matched pair.*

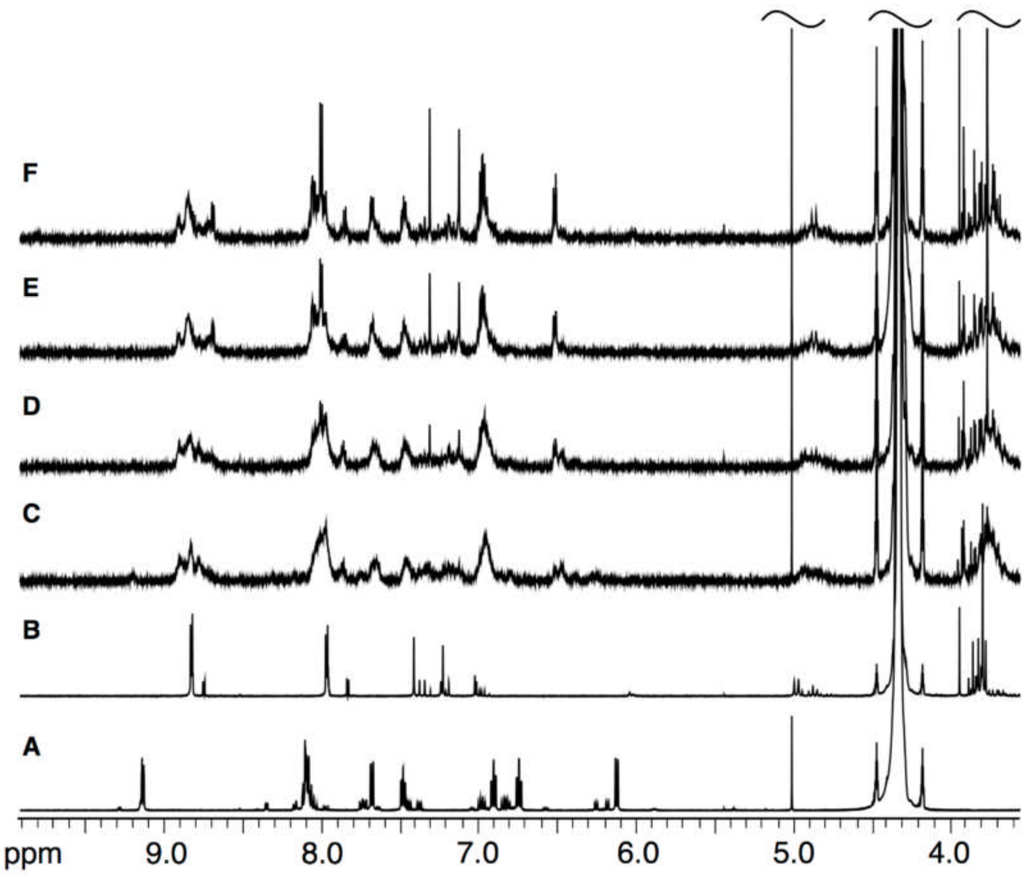

Figure S13. *^{1}H NMR spectra (500 MHz, CD_3NO_2, 298 K, [**F4**] = 2.2 mM) of the complexation of Λ-[Ir(ppy)$_2$(MeCN)$_2$]·BF_4 and one of the enantiomers of tritopic ligand (L), **F4** (A) Λ-[Ir(ppy)$_2$(MeCN)$_2$]·BF_4, (B) **F4**, (C) 10 min, (D) 2 h, (E) 2 days, (F) 6 days. Matched pair.*

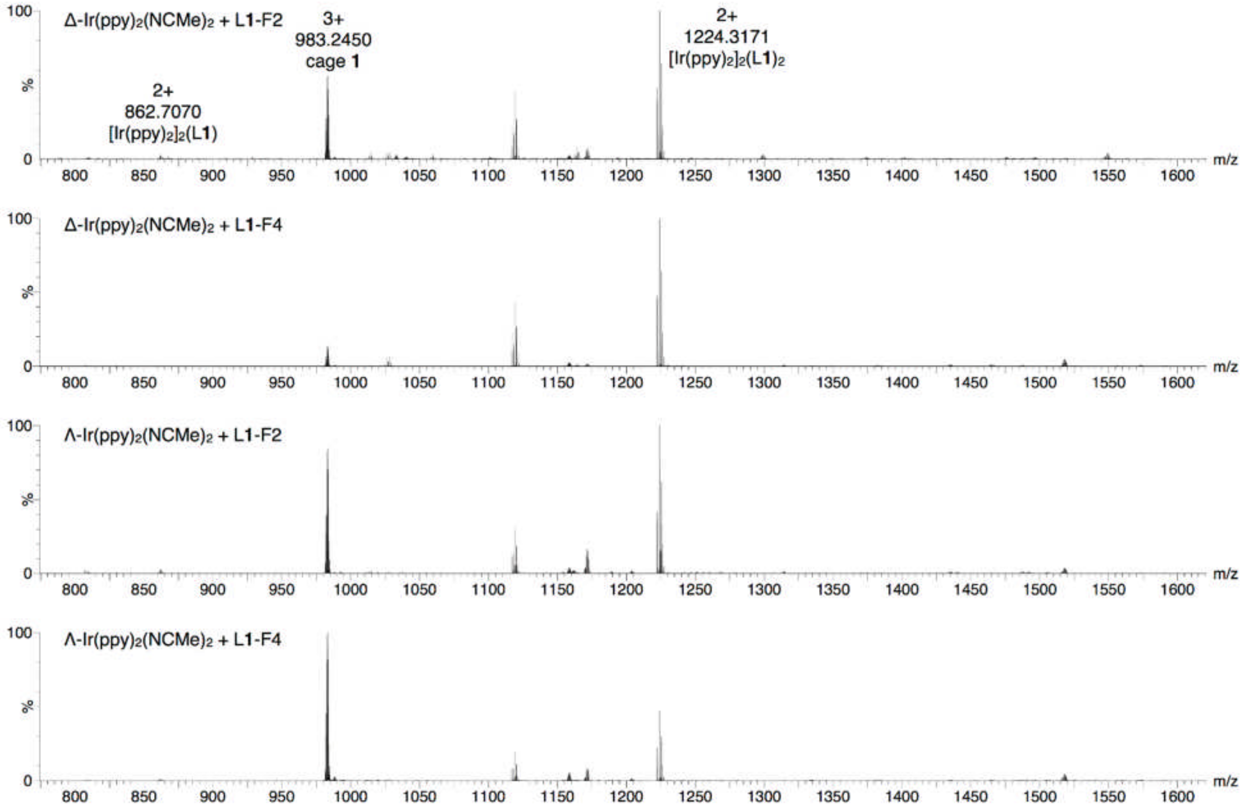

Figure S14: *ESI-MS of pairs of resolved [Ir(ppy)$_2$(MeCN)$_2$]·BF$_4$ and L1.*

{[Ir(ppy)$_2$]$_3$(L2)$_2$}$^{3+}$, cage 2

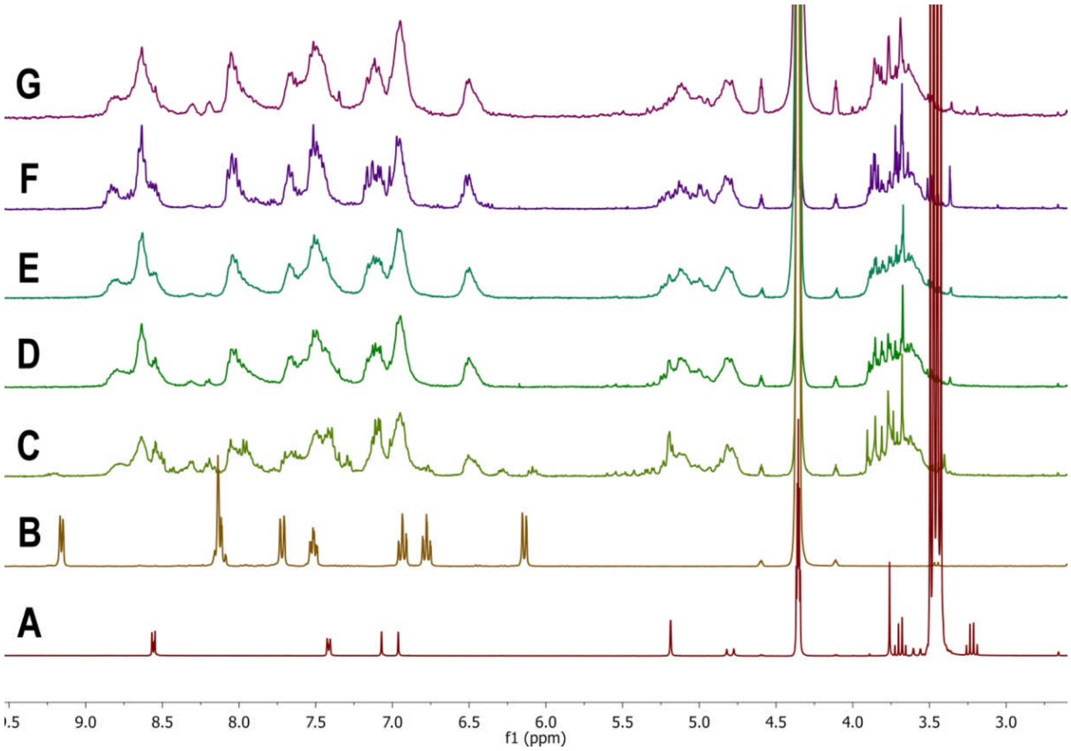

Figure S15: *^{1}H NMR (d_3-MeNO$_2$) following the initial formation of **2**·3PF$_6$ at room temperature. a) (±)-**L2** b), (Δ, Λ)-[Ir(ppy)$_2$(MeCN)$_2$]·PF$_6$, c) immediately after mixing, d) 12hrs RT, e) 48hrs RT, f) 1 week RT, g) Spectrum of re-dissolved powdered **2**·3BF$_4$*

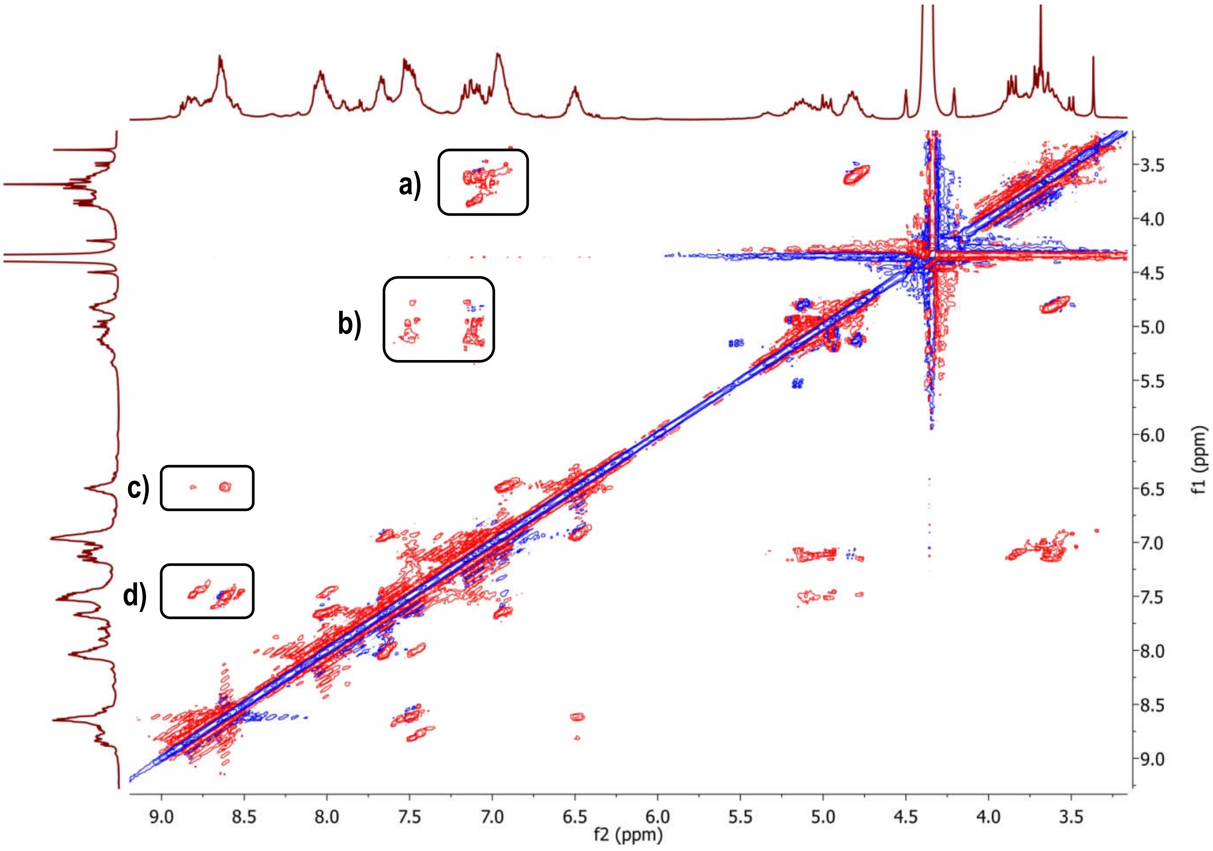

Figure S16: *ROESY spectrum of **2**·3PF$_6$ in d$_3$-MeNO$_2$ solvent, a) coupling between exo/methoxy protons and aryl-CTG protons, b) coupling between CH$_2$ ethyl linker and aryl-CTG protons, as well as the CH$_2$ linker and meta-pyridyl protons c) coupling between H$_{H'}$ on the phenylpyridine ancillary ligand and the ortho-pyridyl proton on **L2**, d) coupling between ortho/meta pyridyl protons and likely inter-ligand phenylpyridine coupling.*

4. MS and NMR of Cage Assembly in the presence of guests

Procedure: L1 (4.0 mgs, 5.53mmol, 2 equivalents) was suspended in deuterated MeNO$_2$ (0.4ml) in an NMR tube. The tube was sonicated for ten minutes and heated (heat gun) until all the material dissolved. A solution of [Ir(ppy)$_2$(MeCN)$_2$] (6.03 mgs, 8.29 mmol, 3 equivalents) and appropriate guest (3 mgs, 19.7mmol, 7.13 equivalents for R/S camphor; 3 mgs, 22.0 mmol, 7.95 equivalents for adamantane; 3 mgs, 12.9 mmol, 4.66 equivalents for R/S Camphor sulfonic acid) in 0.3 mL deuterated MeNO$_2$ was added. An initial spectrum was immediately recorded followed by a subsequent spectrum every 15 minutes up until 2 hours, then at longer intervals.

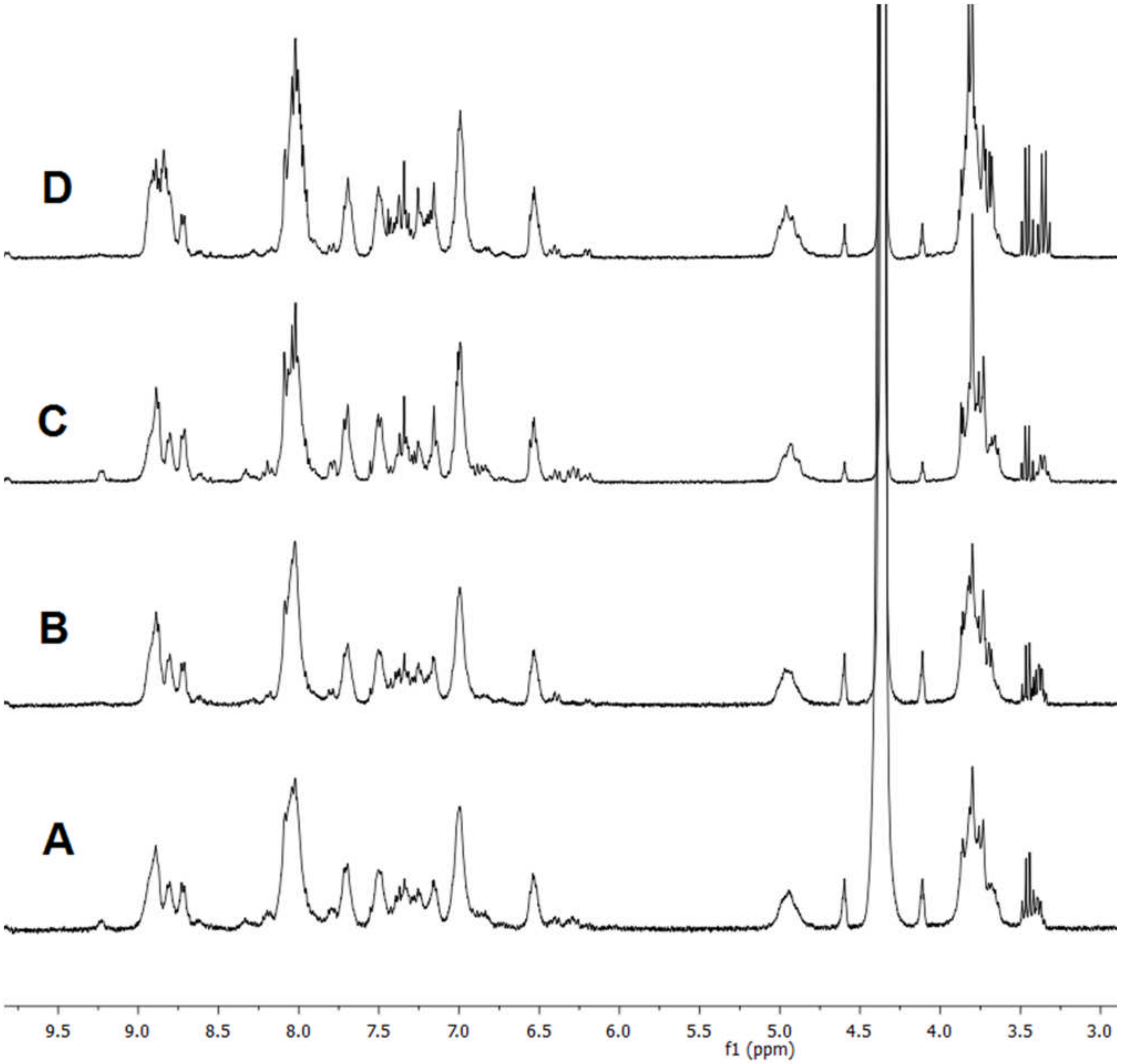

Figure S17: *Section of* ^{1}H *NMR (300 MHz. d_3-$MeNO_2$) of 3:2 mixture of (Δ, Λ)-[Ir(ppy)$_2$(MeCN)$_2$]·PF_6 and (±)-**L2** with A: adamantane after 48 hrs equilibration; B: no added guest after 26 hrs; C: added S-camphor after 26 hrs equilibration; D: added R-camphor after 26 hrs equilibration. Even after almost double the equilibration time the sample with admanantane is less sorted than those with chiral guests.*

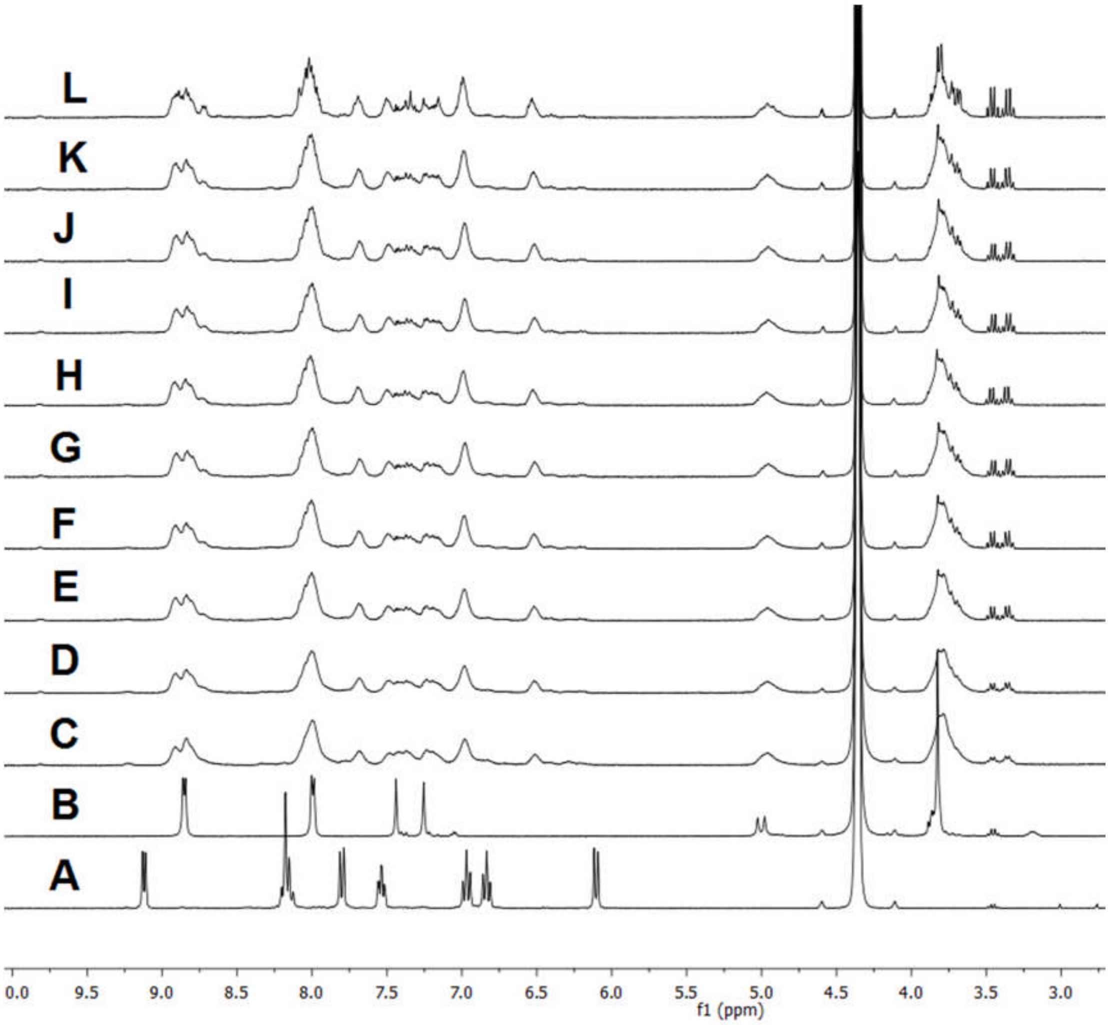

(a)

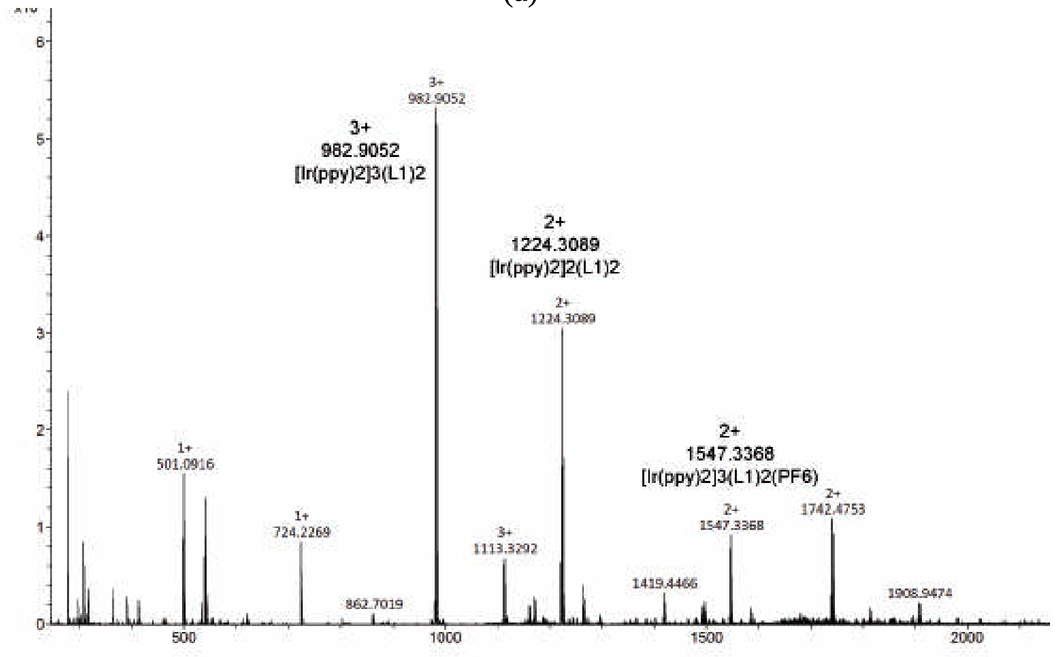

(b)

Figure S18: *3:2 mixture of (Δ, Λ)-[Ir(ppy)$_2$(MeCN)$_2$]·PF$_6$ and (±)-**L2** with excess of R-camphor $C_{10}H_{16}O$ added. (a) ^{1}H NMR (300 MHz. d$_3$-MeNO$_2$) of initial cage **1** formation A = (Δ,Λ)-[Ir(ppy)$_2$(MeCN)$_2$]·PF$_6$; B= (±)-**L1**; C = initial: D = 15 mins; E = 30 mins; F = 45 mins; G = 60 mins; H = 75 mins; I = 90 mins; J = 105 mins; K = 120 mins; L = 26 hrs. (b) ESI-MS*

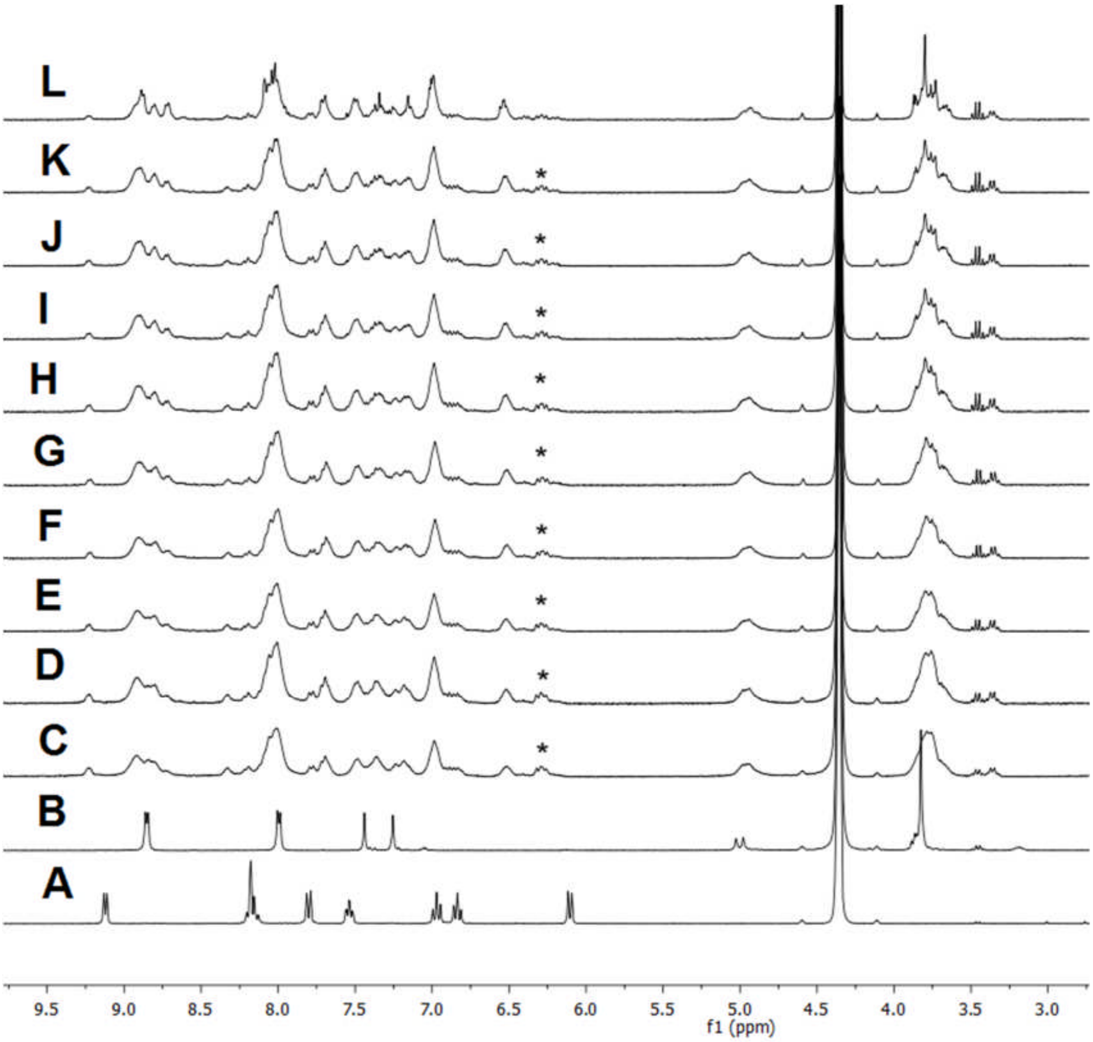

(a)

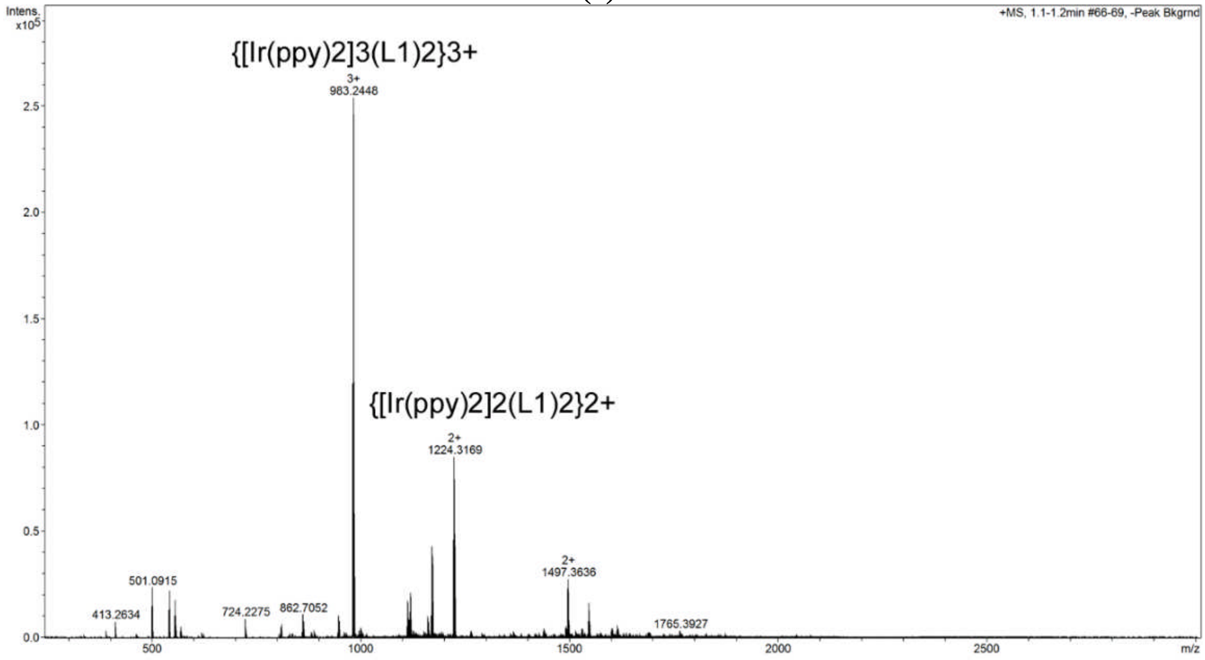

(b)

Figure S19: *3:2 mixture of (Δ, Λ)-[Ir(ppy)$_2$(MeCN)$_2$]·PF$_6$ and (±)-**L2** with excess S-camphor C$_{10}$H$_{16}$O added. (a) ^{1}H NMR (300 MHz. d$_3$-MeNO$_2$) of initial cage **1** formation * indicates unbound [Ir(ppy)$_2$]$^+$ A = (Δ,Λ)-[Ir(ppy)$_2$(MeCN)$_2$]·PF$_6$; B= (±)-**L1**; C = initial: D = 15 mins; E = 30 mins; F = 45 mins; G = 60 mins; H = 75 mins; I = 90 mins; J = 105 mins; K = 120 mins; L = 26 hrs. (b) ESI-MS after 3 days.*

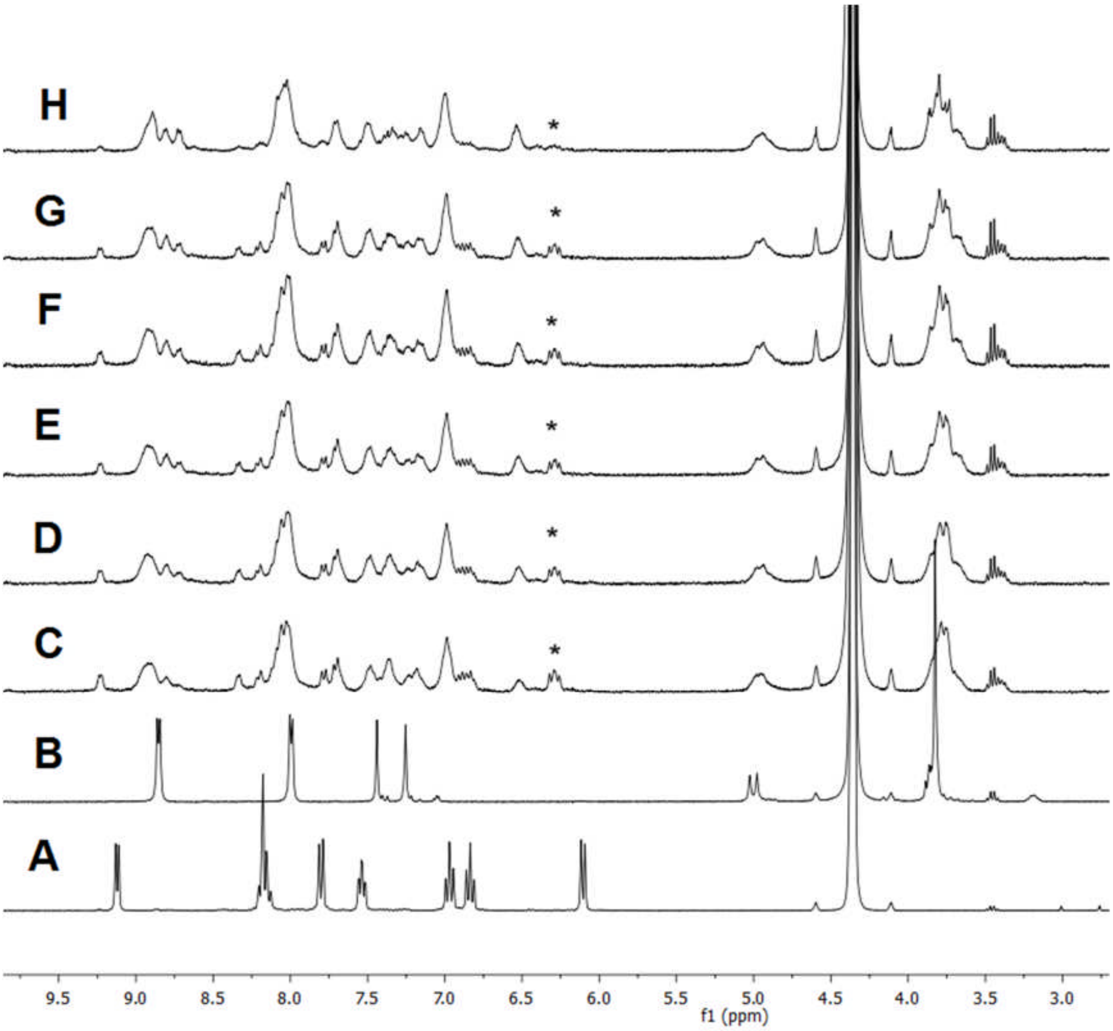

(a)

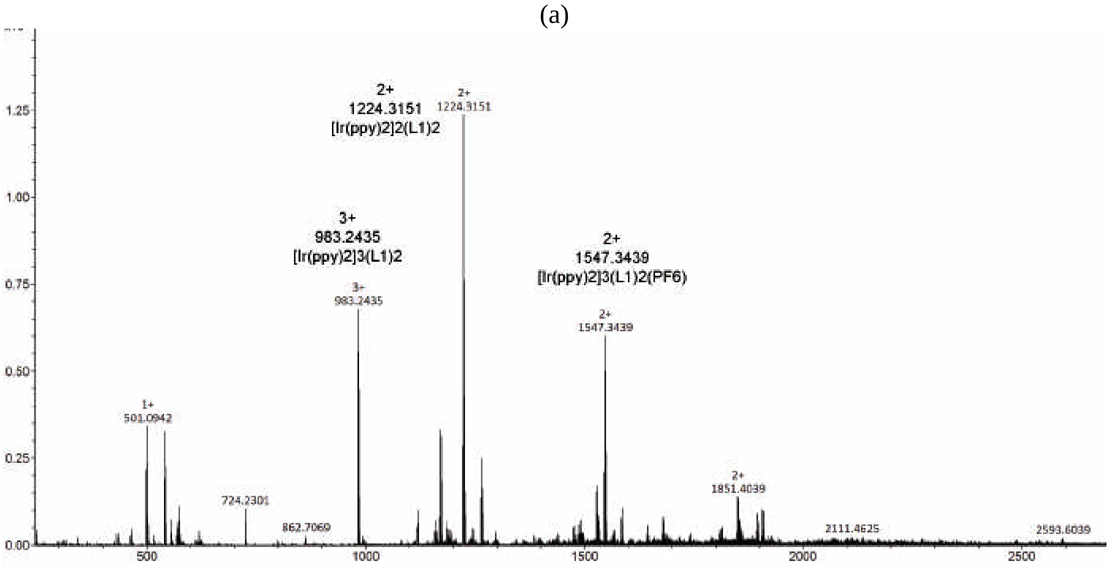

(b)

Figure S20: *3:2 mixture of (Δ, Λ)-[Ir(ppy)$_2$(MeCN)$_2$]·PF$_6$ and (±)-**L2** with excess adamantane $C_{10}H_{16}$ added. (a) ^{1}H NMR (300 MHz. d$_3$-MeNO$_2$): formation of cage **1** is slower than observed in the absence of adamantane (cf. Figure S7 for similar experiment with no adamantane added). * indicates unbound [Ir(ppy)$_2$]$^+$. A = (Δ,Λ)-[Ir(ppy)$_2$(MeCN)$_2$]·PF$_6$; B= (±)-**L1**; C = initial: D = 30 mins; E = 60 mins; F = 90 mins; G = 120 mins; H = 48 hrs. (b) ESI-MS after 48 hours.*

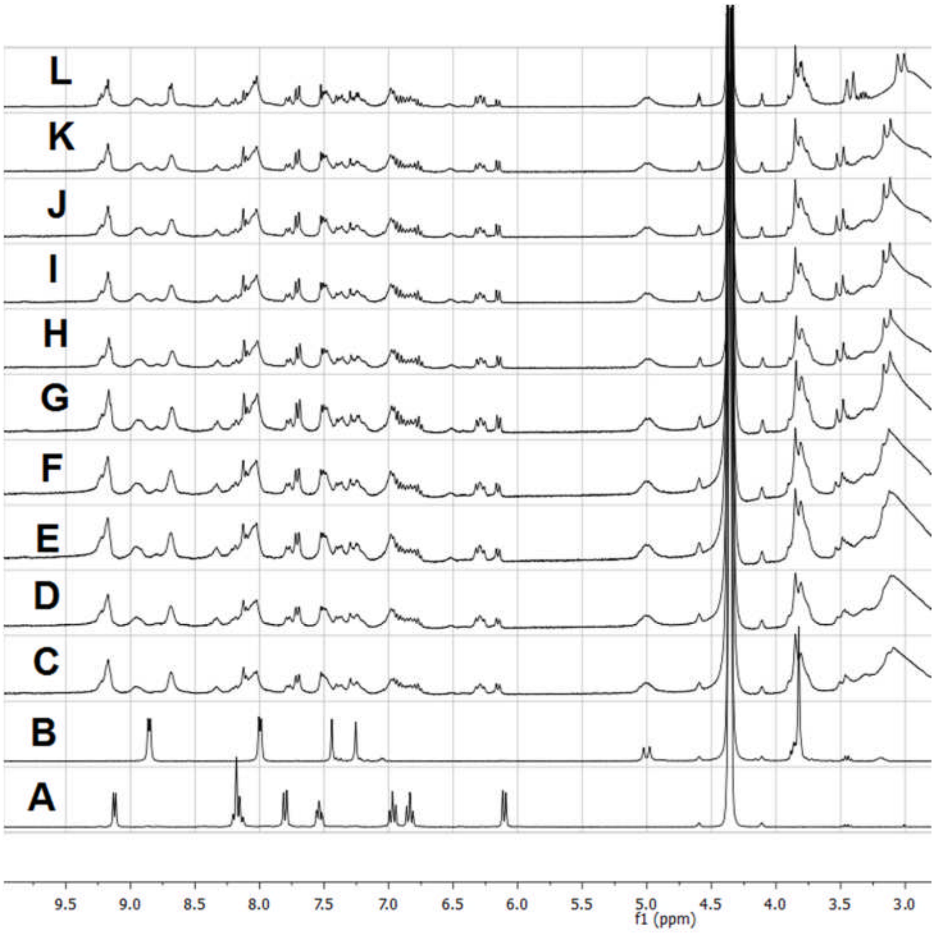

(a)

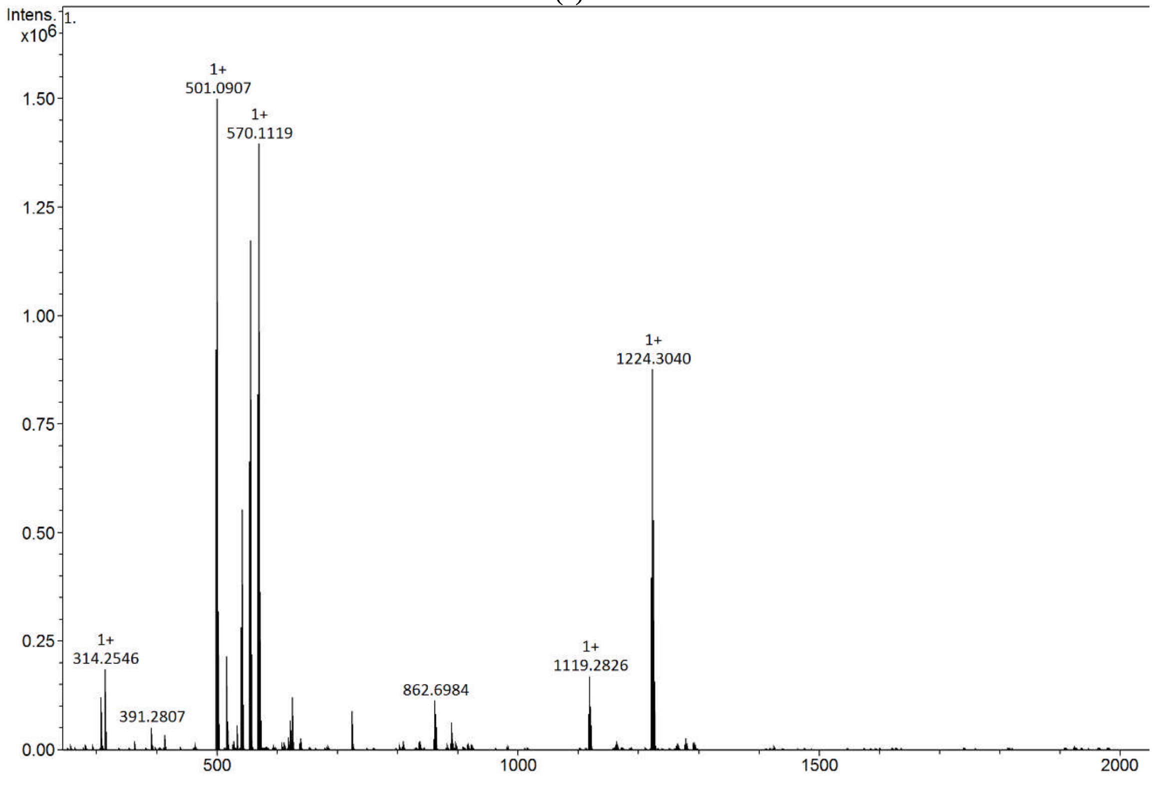

(b)

Figure S21: *3:2 mixture of (Δ, Λ)-[Ir(ppy)$_2$(MeCN)$_2$]·PF$_6$ and (±)-**L2** with excess of R-camphor-sulfonic acid added. Cage **1** formation is prevented.^{1}H NMR (300 MHz. d$_3$-MeNO$_2$) A = (Δ,Λ)-[Ir(ppy)$_2$(MeCN)$_2$]·PF$_6$; B= (±)-**L1**; C = initial: D = 15 mins; E = 30 mins; F = 45 mins; G = 60 mins; H = 75 mins; I = 90 mins; J = 105 mins; K = 120 mins; L = 48 hrs. (b) ESI-MS after 48 hrs.*

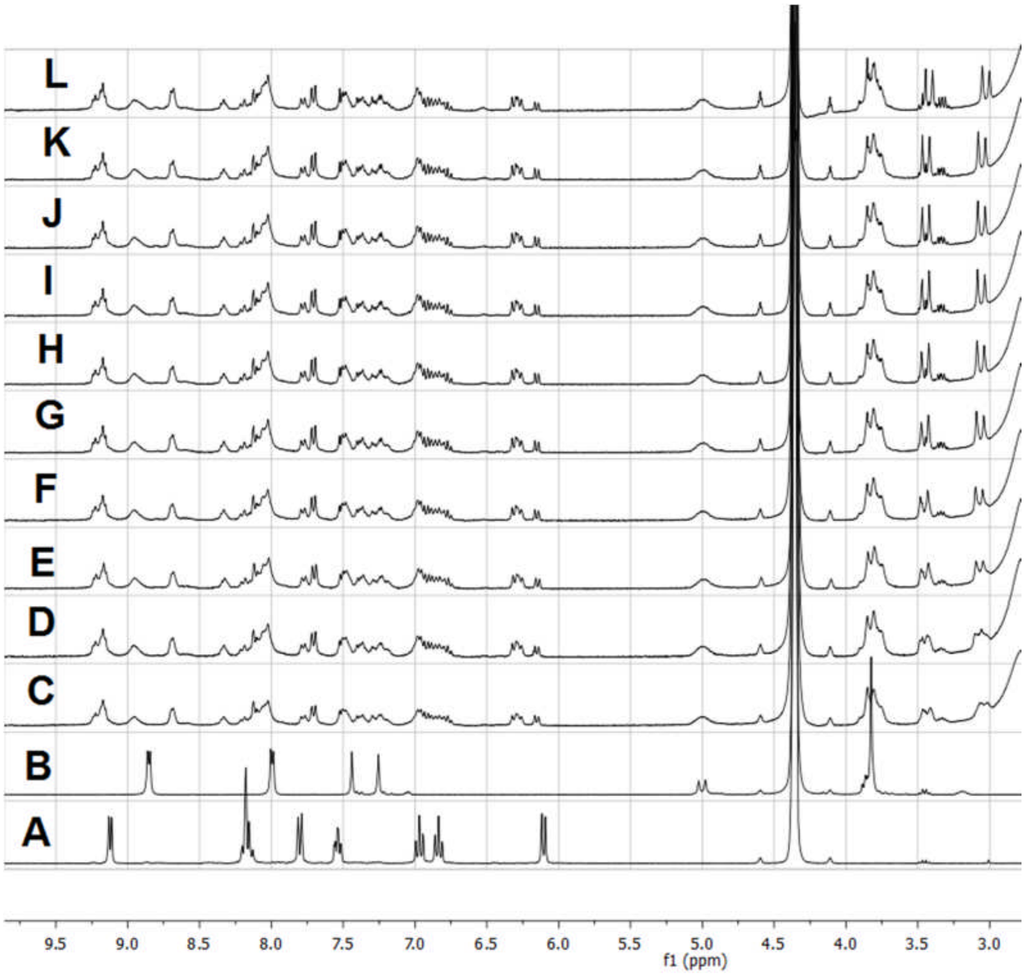

(a)

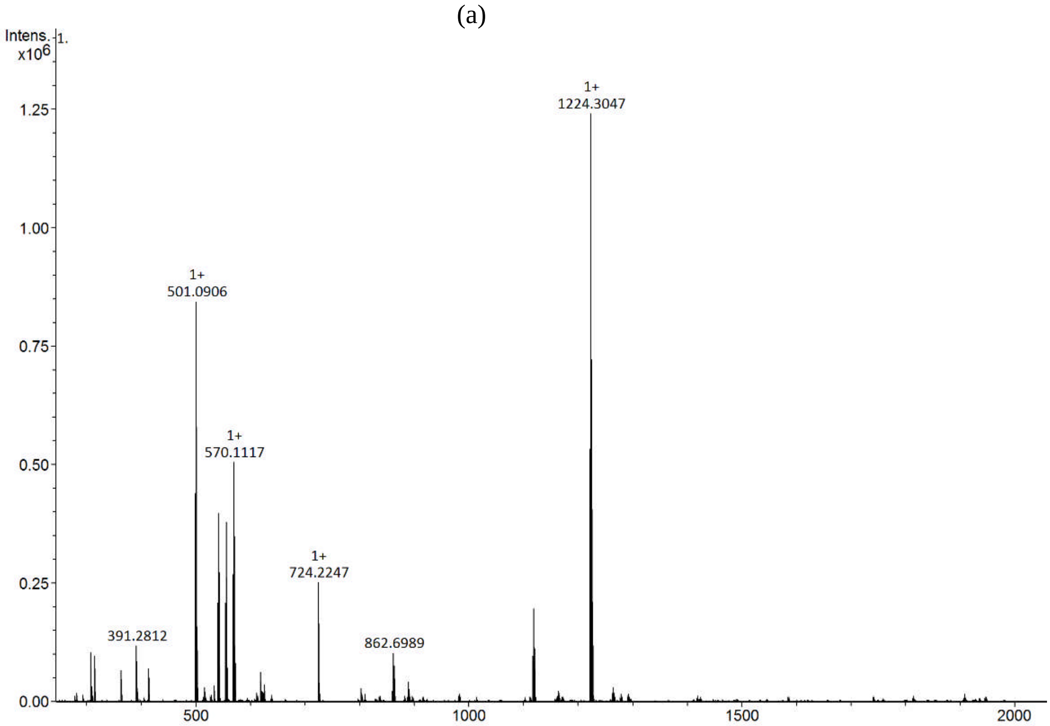

(b)

Figure S22: *3:2 mixture of (Δ, Λ)-[Ir(ppy)$_2$(MeCN)$_2$]·PF$_6$ and (±)-**L2** with excess of S-camphor-sulfonic acid (left) and S-camphor-sulfonic acid (right) added. In both cases cage **1** formation is prevented.^{1}H NMR (300 MHz. d$_3$-MeNO$_2$) A = (Δ,Λ)-[Ir(ppy)$_2$(MeCN)$_2$]·PF$_6$; B= (±)-**L1**; C = initial: D = 15 mins; E = 30 mins; F = 45 mins; G = 60 mins; H = 75 mins; I = 90 mins; J = 105 mins; K = 120 mins; L = 48 hrs. (b) ESI-MS after 48 hrs.*

5. X-ray Crystallography

A crystal of complex **1**·$3BF_4$·n($MeNO_2$) was mounted under inert oil on a MiTeGen tip and flash frozen to 100(1) K using an OxfordCryosystems low temperature device. X-ray diffraction data were collected using Cu-K_α radiation (λ= 1.54184 Å) using an Agilent Supernova dual-source diffractometer with Atlas S2 CCD detector and fine-focus sealed tube generator. Data were corrected for Lorenztian and polarization effects and absorption corrections were applied using multi-scan methods. The structures were solved by direct methods using SHELXS-97 and refined by full-matrix on F^2 using SHELXL-97.[6] Crystals were very poorly diffracting due to high levels of solvation and disordered counter-anions. Most high angle data was unobserved. While the cage framework and some solvent nitromethane positions were located in the difference map and included in the refinement, the BF_4^- counter-anions were not located and the true degree of solvation is likely to be significantly higher than was determined crystallographically. Counter-anions were included in the molecular formula, but not missing solvent. The large void spaces and diffuse nature of residual electron density meant that the SQUEEZE routine of PLATON was employed.[7] Fifteen of the phenyl or pyridyl groups were refined with rigid body constraints. Only the Ir and ordered parts of the CTG-type ligands were refined anisotropically and global restraints were employed on anisotropic displacing parameters. One isonicotinoyl group was refined as being disordered across two positions, each at 0.5 occupancy. Two phenyl-pyridyl groups and one isonicotinoyl groups were each refined with a group isotropic displacement parameter. Nine inter-atomic distances (for Ir-C/N or C-C bonds of phenyl-pyridines) were restrained to be chemically reasonable.

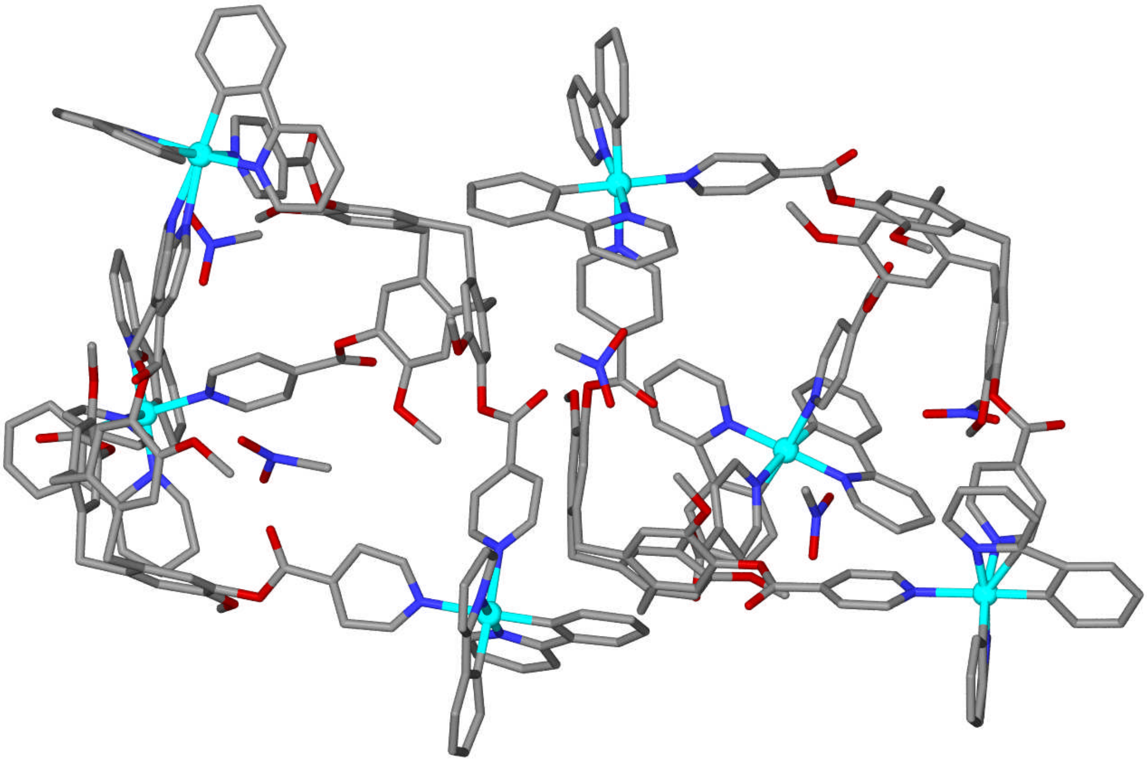

***Figure S23**: Asymmetric unit of the crystal structure of **1**·$3BF_4$·n(CH_3NO_2), hydrogen atoms omitted for clarity.*

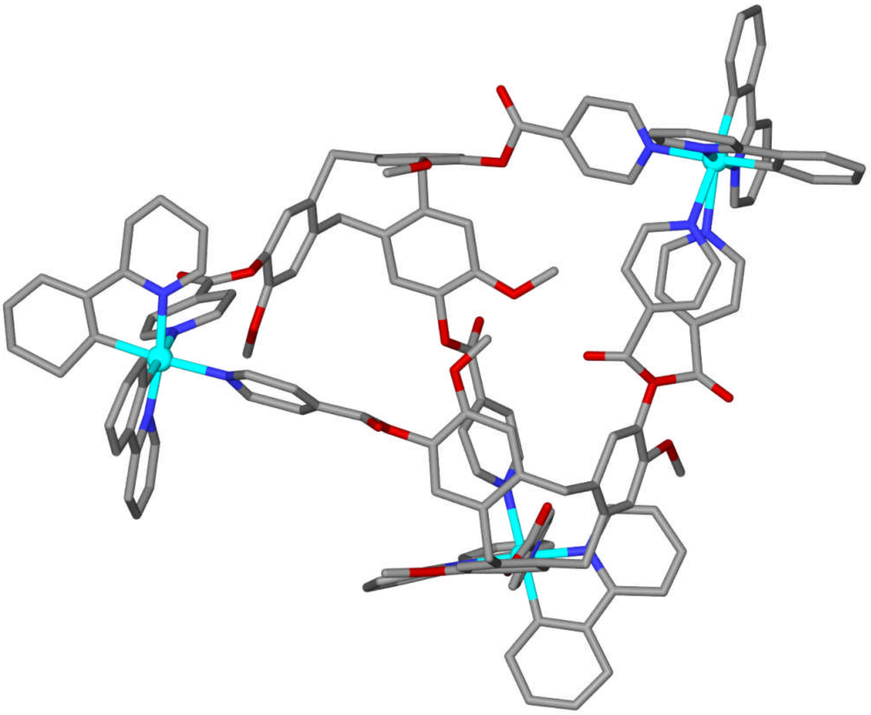

(a)

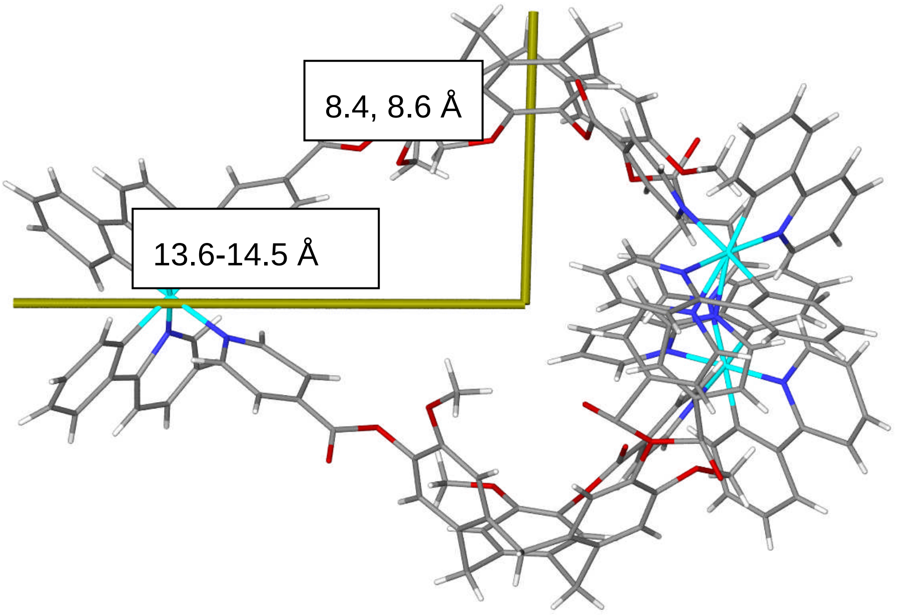

(b)

Figure S24: *Metallo-cryptophane structures from crystal structure of* **1**$\cdot$*3BF*$_4$$\cdot$*n(CH*$_3$*NO*$_2$*). (a) Crystallographically distinct cage from that shown in manuscript, highlighting disorder of one isonicotinoyl group; (b) cage with sizes of both cages indicated.*

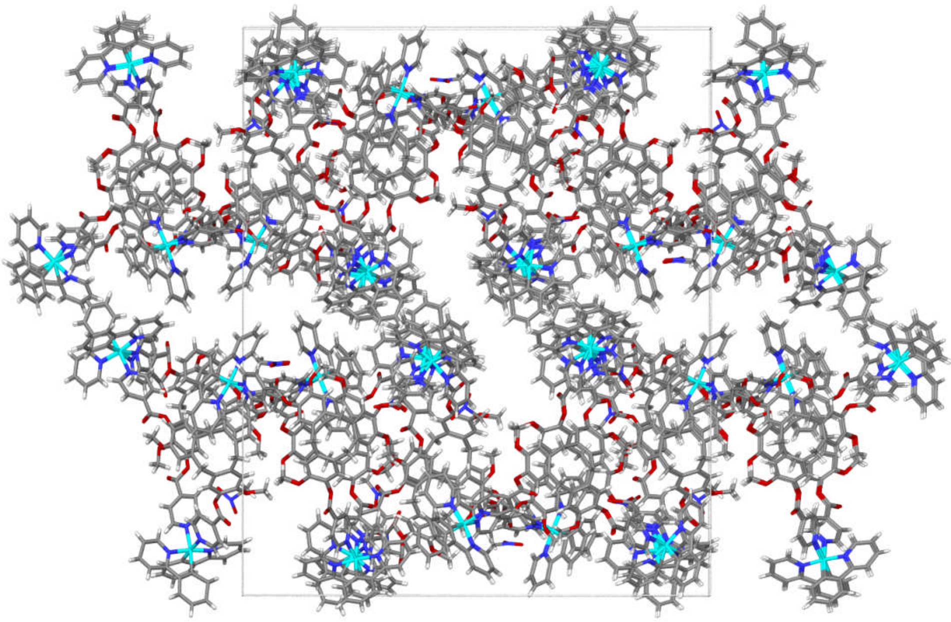

(a)

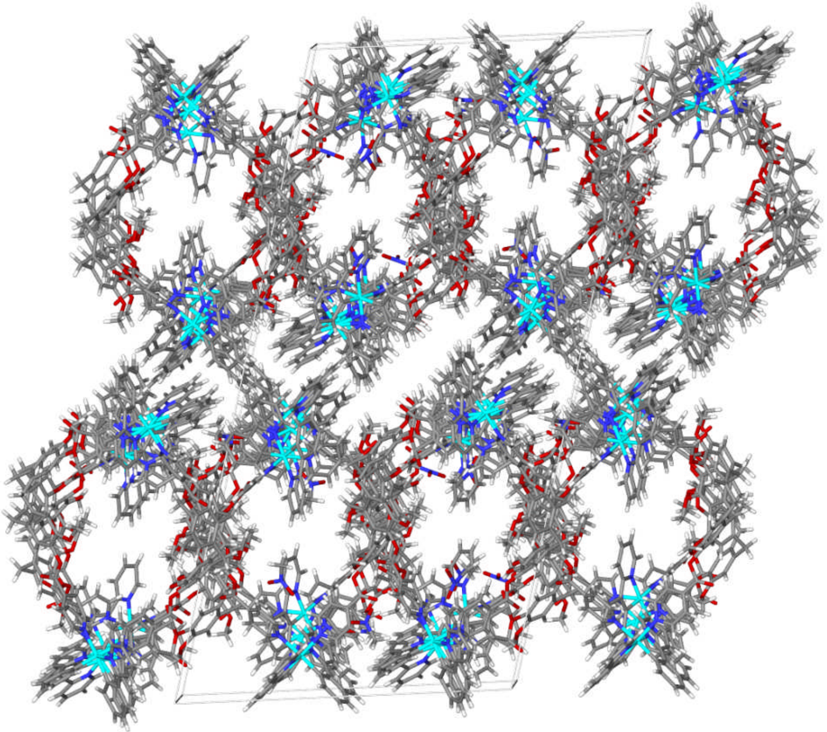

(b)

Figure S25: *Unit cell diagrams from crystal structure of* ***1***$\cdot$*$3BF_4\cdot n(CH_3NO_2)$; (a) viewed down a; (b) viewed down b.*

6. Photophysical Studies

All samples were prepared in HPLC grade dichloromethane with varying concentrations in the order of 10^{-4} – 10^{-6} M. Absorption spectra were recorded at room temperature using a Shimadzu UV-1800 double beam spectrophotometer. Molar absorptivity determination was verified by linear least-squares fit of values obtained from at least four independent solutions at varying concentrations with absorbance ranging from 6.05×10^{-5} to 2.07×10^{-5} M.

The sample solutions for the emission spectra were prepared in HPLC-grade DCM and degassed *via* freeze-pump-thaw cycles using a quartz cuvette designed in-house. Steady-state emission and excitation spectra and time-resolved emission spectra were recorded at 298 K using an Edinburgh Instruments F980. All samples for steady-state measurements were excited at 360 nm, while samples for time-resolved measurements were excited at 378 nm using a PDL 800-D pulsed diode laser. Emission quantum yields were determined using the optically dilute method.[8] A stock solution with absorbance of *ca*. 0.5 was prepared and then four dilutions were prepared with dilution factors between 2 and 20 to obtain solutions with absorbances of *ca*. 0.095 0.065, 0.05 and 0.018, respectively. The Beer-Lambert law was found to be linear at the concentrations of these solutions. The emission spectra were then measured after the solutions were rigorously degassed *via* three freeze-pump-thaw cycles prior to spectrum acquisition. For each sample, linearity between absorption and emission intensity was verified through linear regression analysis and additional measurements were acquired until the Pearson regression factor (R^2) for the linear fit of the data set surpassed 0.9. Individual relative quantum yield values were calculated for each solution and the values reported represent the slope value. The equation $\Phi_s = \Phi_r(A_r/A_s)(I_s/I_r)(n_s/n_r)^2$ was used to calculate the relative quantum yield of each of the sample, where Φ_r is the absolute quantum yield of the reference, *n* is the refractive index of the solvent, *A* is the absorbance at the excitation wavelength, and *I* is the integrated area under the corrected emission curve. The subscripts s and r refer to the sample and reference, respectively. A solution of quinine sulfate in 0.5 M H_2SO_4 (Φ_r = 54.6%)[9] was used as external references.[10]

PMMA doped films were prepared by spin coating the samples from a solution of 2-methoxyethanol (HPLC grade) containing 5 % w/w of the desired sample. Steady-state emission and excitation spectra and time-resolved emission spectra of both powders and doped films were recorded at 298 K using an Edinburgh Instruments F980. Solid-state PLQY measurements of thin films were performed in an integrating sphere under a nitrogen purge in a Hamamatsu C9920-02 luminescence measurement system.[11]

Table **S1**. UV-Vis spectroscopy.

cage	UV-Vis (nm) [ε(×10^3 M^{-1} cm^{-1})][a]
**1**	260 [85.7], 269 [82.9], 309 [29.9], 342 [19.5], 379 [11.9], 419 [7.2], 471 [2.5]
**2**	254 [92.8], 269 [82.9], 294 [63.5], 342 [18.5], 385 [9.1], 416 [5.6], 474 [1.3]

[a] Measurements in DCM at 298 K

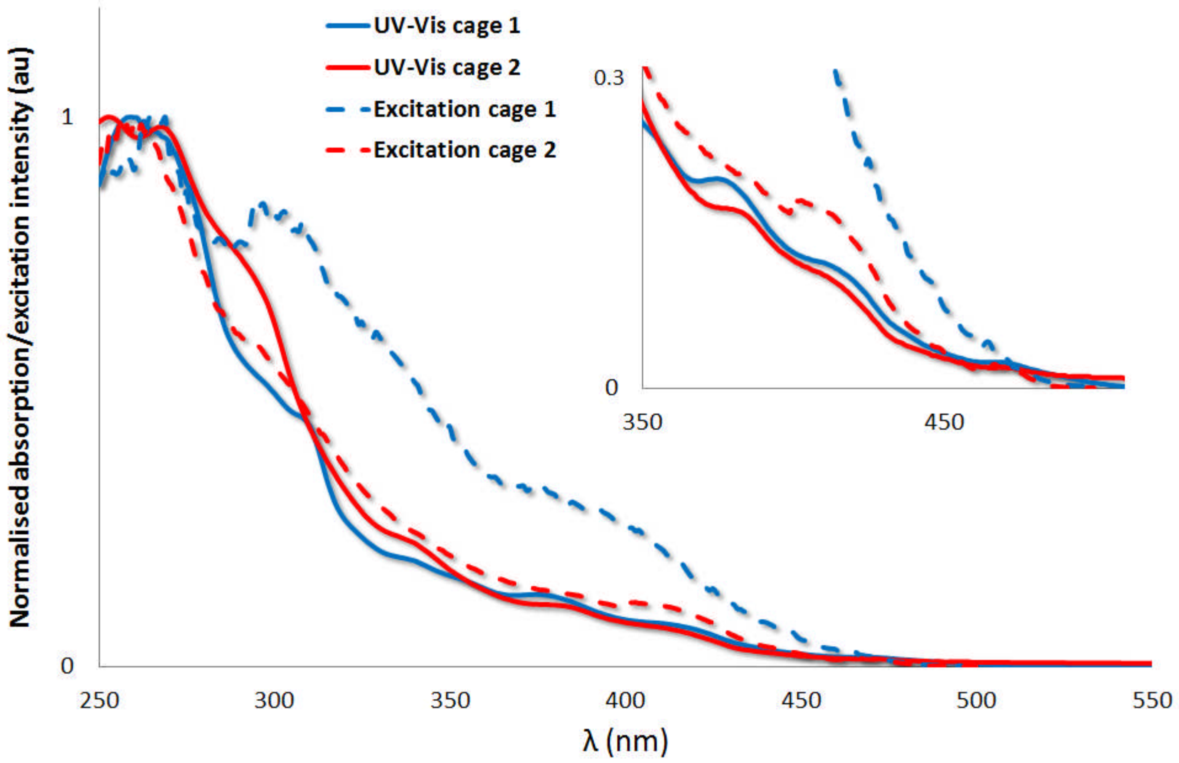

Figure S26*. UV-Vis spectra of cage* ***1,*** *in red solid line and cage* ***2,*** *in light blue solid line and excitation spectra of cage* ***1****, in red dashed line and cage* ***2****, in light-blue dashed line collected in* CD_2Cl_2 *at 298 K.*

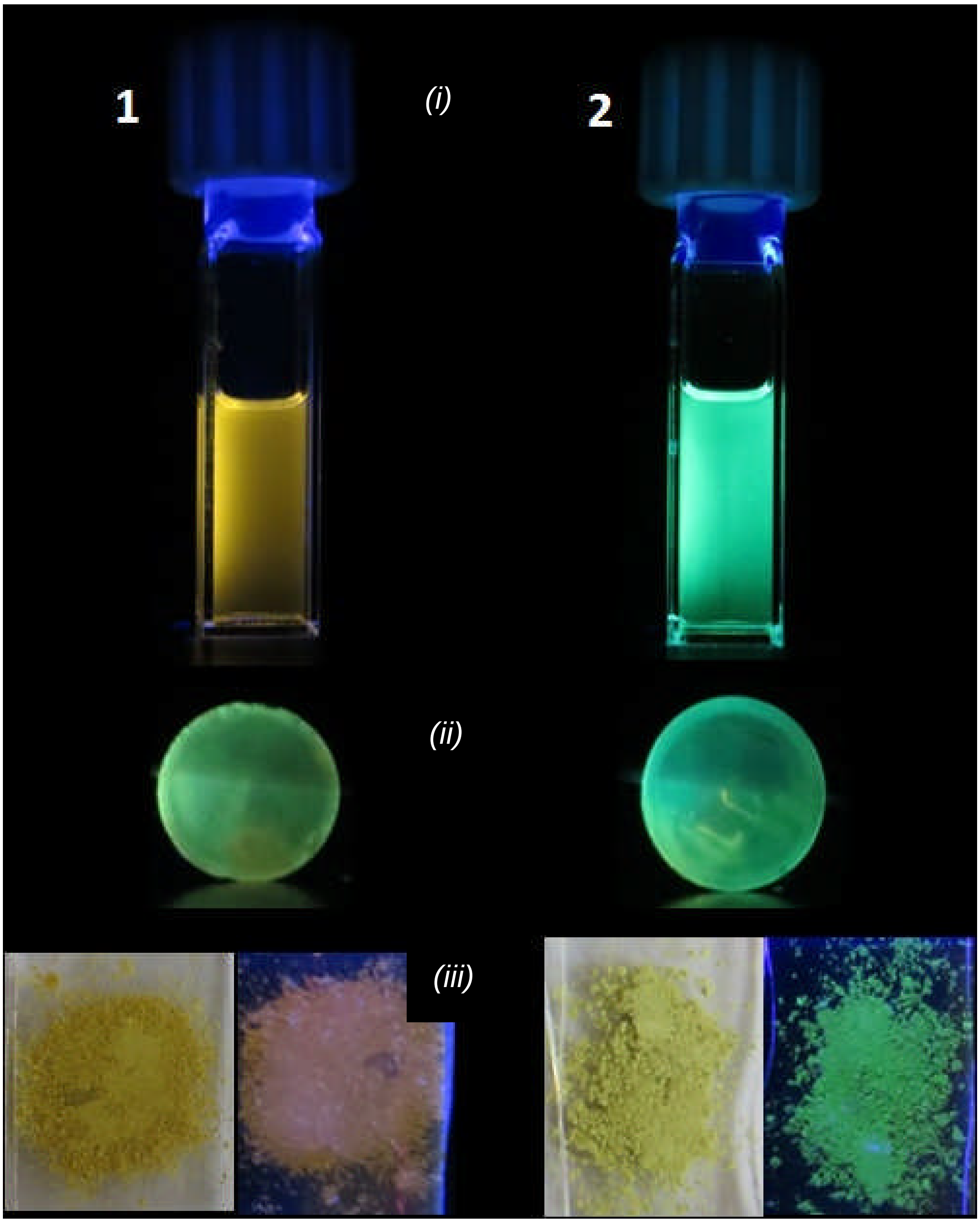

(a)

(i) 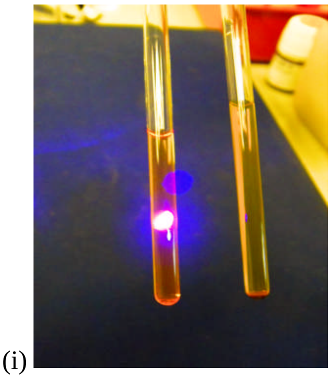

(ii) 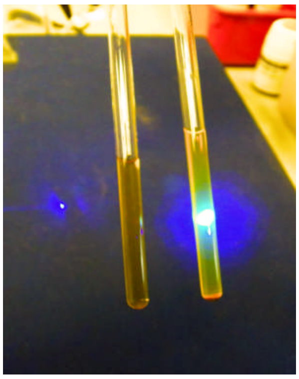

Figure S27. *Images of cage* ***1*** *and cage* ***2****: (a) (i) dark room image on irradiation of DCM solution, (ii) dark room image on irradiation of PMMA doped films; (iii) powder form under natural light (left image) and on irradiation (right image). (b)* CD_3NO_2 *solution with irradiation using 405 nm laser pen, (i) cage* ***1****; (ii) cage* ***2****.*

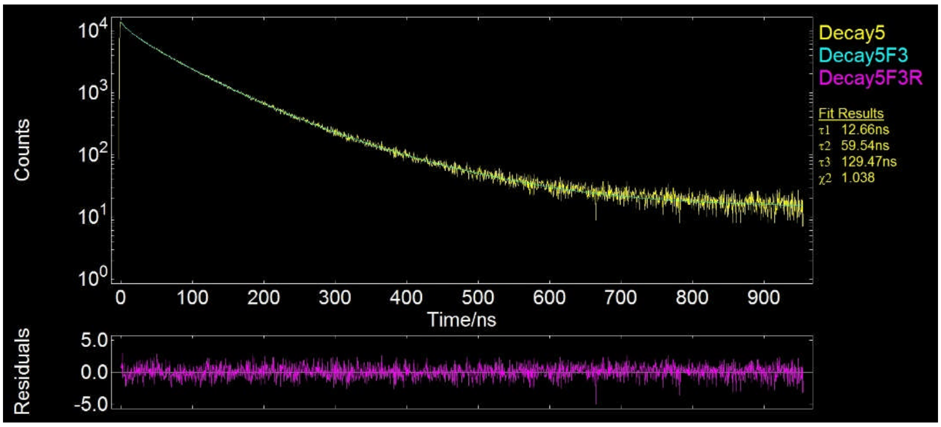

Figure S28. *Lifetime decays of* **1** *after excitation at 379 nm in degassed DCM at 298 K*

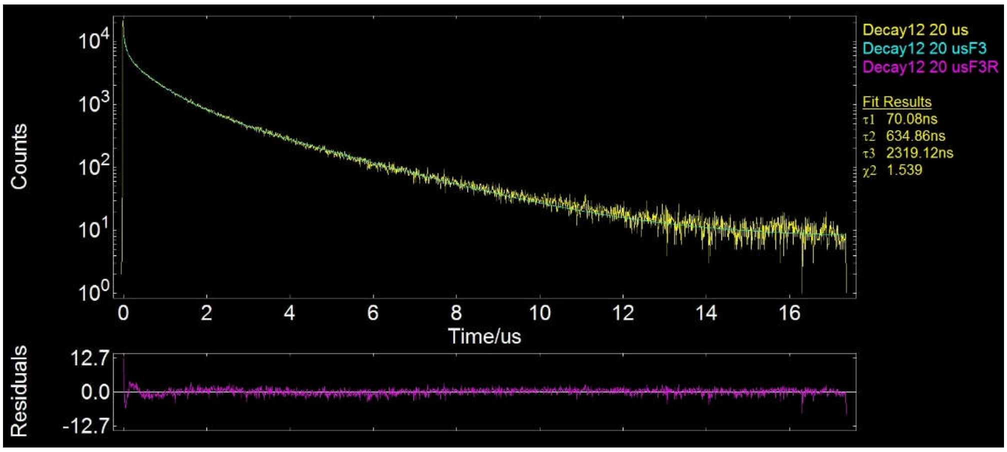

Figure S29. *Lifetime decays of* **1** *after excitation at 379 nm in a PMMA doped film at 298 K.*

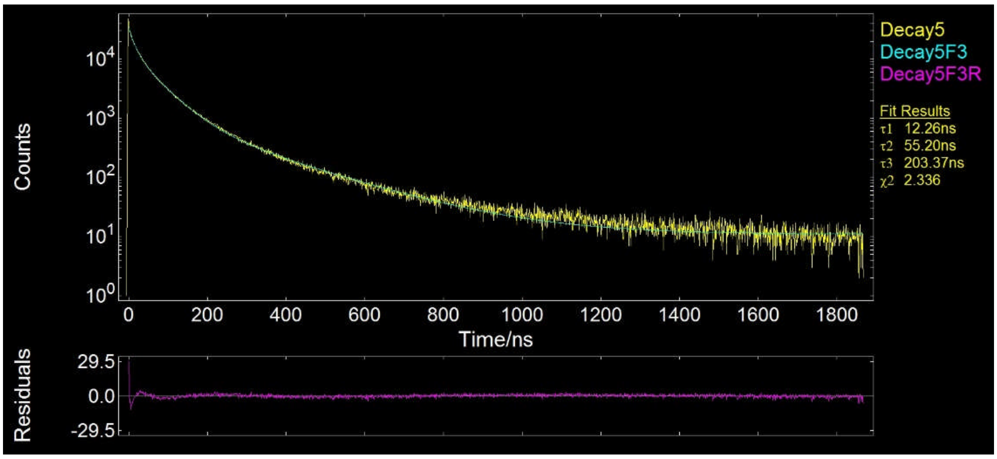

Figure S30. *Powder Lifetime decays of* **1** *after excitation at 379 nm at 298 K.*

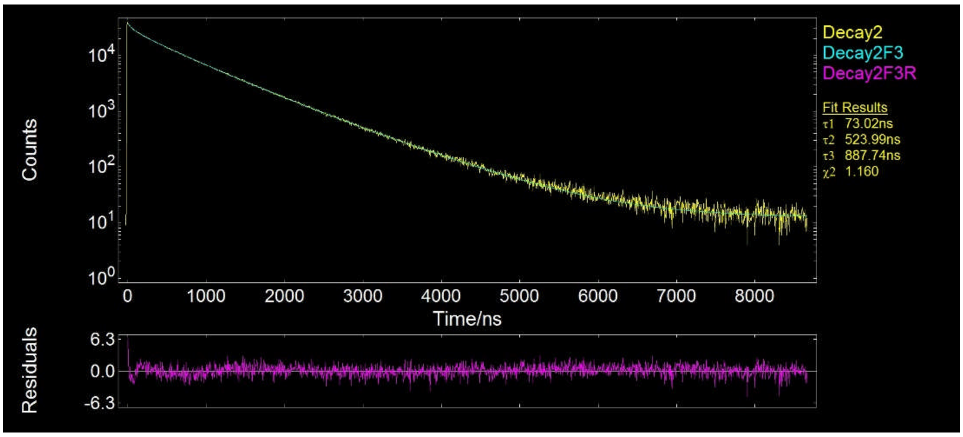

Figure S31. *Lifetime decays of* ***2*** *after excitation at 379 nm in degassed DCM at 298 K.*

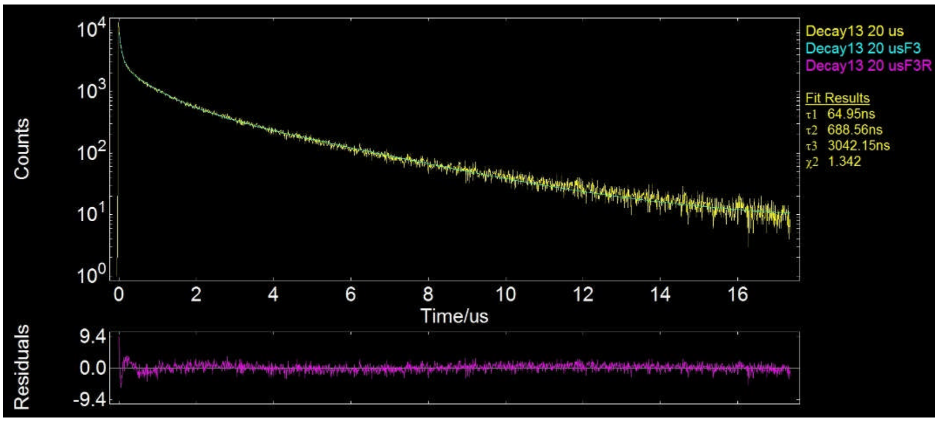

Figure S32. *Lifetime decays of* ***2*** *after excitation at 379 nm in a PMMA doped film at 298 K.*

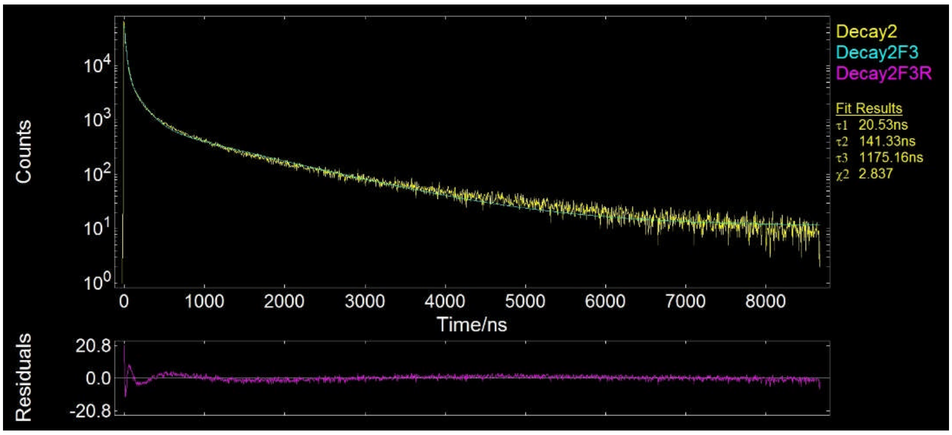

Figure S33. *Powder Lifetime decays of* ***2*** *after excitation at 379 nm at 298 K.*

PMMA repeat experiments and degradation experiments

Following the condition reported in the manuscript cage **1** and **2** (doped in PMMA, 10% of cages) were spin-coated on quartz substrates at increased cage concentration to aid characterisation. The

emission spectra and emission lifetimes of the two doped-films are identical to that reported in the manuscript (the emission spectra are illustrated in Figure S27). This indicates that the bulk materials are stable after months at room temperature. In addition, after spin-coating the samples multiple times, identical films are formed on the quartz substrates.

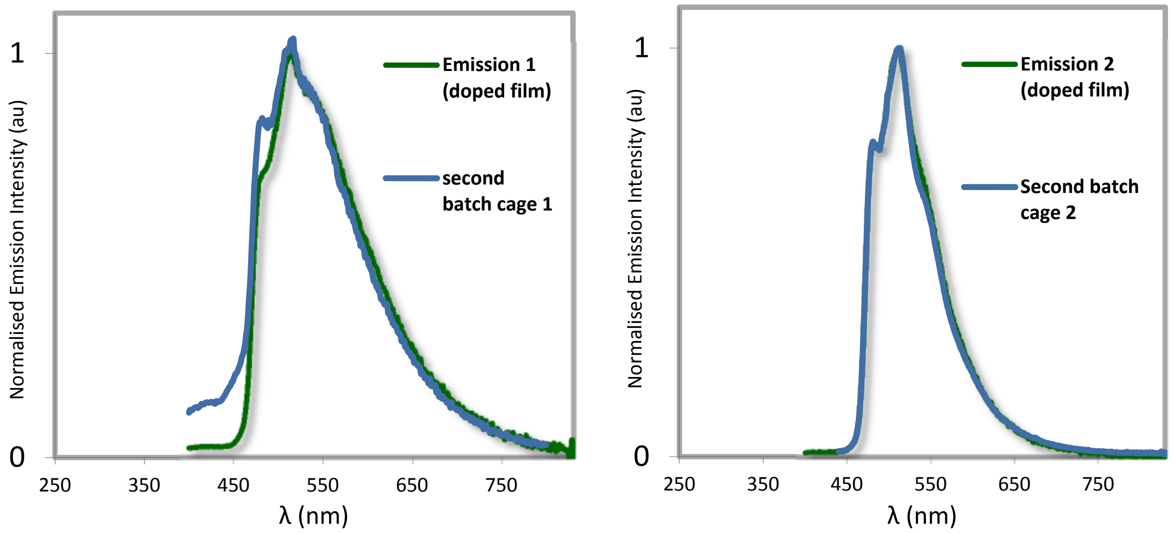

***Figure S34**. Normalised photoluminescence spectra of PMMA doped films with 5 wt % (in green) or 10% (in blue) of cages spin-coated on a quartz substrate. For both cages **1** and **2** the two emissions have been collected from two separated batches at two different period of time leading the same emission spectra.*

The films of both cages were then dissolved in CD_2Cl_2 and ^{1}H NMR spectra were collected. The solubility of cage **1** in CD_2Cl_2 is very low and consequently the resolution of the ^{1}H-NMR was poor, a slightly better-resolved NMR was collected for cage **2** (Figure S28). In this case it can be noted that very similar NMR spectra of the neat cage **2** and PMMA-doped cage 2 (after spin-coating) are observed. This is a good indication that the cages are still intact after their spin-coating deposition on the quartz substrate. Note: the PMMA-doped films contains less than 1 mg of cages (90% is PMMA) and therefore highly-resolution NMRs of these systems cannot be collected.

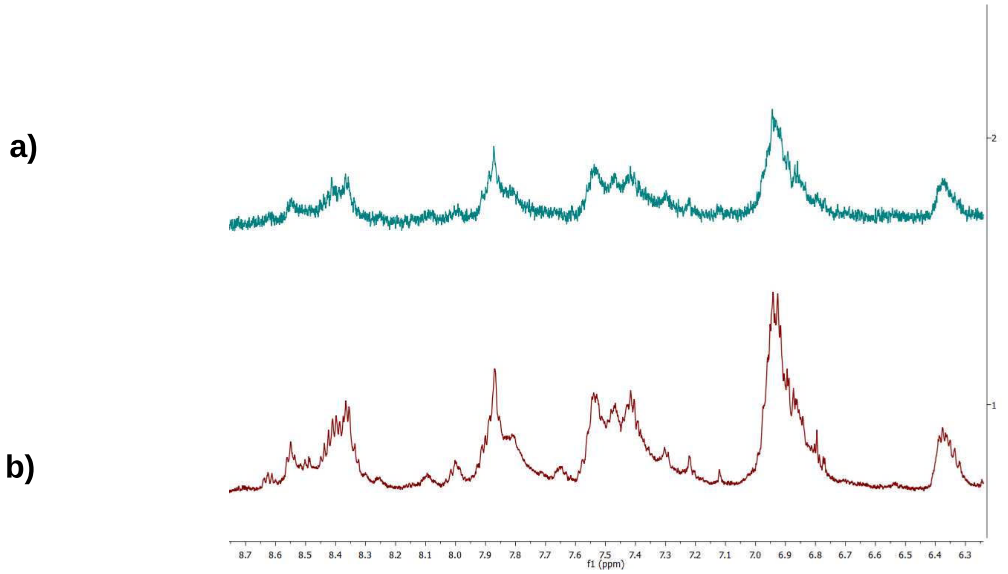

***Figure S35**. Aromatic region of the ^{1}H NMR spectra of a) PMMA-doped cage **2** after being spin-coated and re-dissolved in CD_2Cl_2 and b) neat cage **2** in CD_2Cl_2.*

7. References

1. M. J. Hardie, C. J. Sumby, *Inorg. Chem.*, **2004**, *43*, 6872-6874

2. T. K. Ronson, C. Carruthers, J. Fisher, T. Brotin, L. P. Harding, P. J. Rizkallah, M. J. Hardie, *Inorg. Chem.*, **2010**, *49*, 675-685.

3. K. A. McGee, K. R. Mann, *Inorg. Chem.* **2007**, *49*, 7800-7809.

4. a) D. R. Martir, C. Momblona, A. Pertegas, D. B. Cordes, A. M. Z. Slawin, H. J. Bolink, E. Zysman-Colman, *ACS Appl. Mater. Interfaces* **2016**, *8*, 33907; b) A. M. Bünzli, E. C. Constable, C. E. Housecroft, A. Prescimone, J. A. Zampese, G. Longo, L. Gil-Escrig, A. Pertegás, E. Ortí and H. J. Bolink, *Chem. Sci.* **2015**, *6*, 2843.

5. M. Nilsson, *J. Mag. Resonance*, **2009**, *200*, 296-302.

6. G. M. Sheldrick, *Acta Cryst.* **2008**, *A64*, 112-122.

7. A. L. Spek, *Acta Cryst.*, **1990**, *A46*, C34.

8. G. A. Crosby, J. N. Demas, *J. Phys. Chem.*, **1971**, *75*, 991-1024.

9. W. H. Melhuish, *J. Phys. Chem.*, **1961**, *65*, 229-235.

10. A. M. Brouwer, *Pure Appl. Chem.*, **2011**, *83*, 2213-2228.

11. N. C. Greenham, I. D. W. Samuel, G. R. Hayes, R. T. Phillips, Y. A. R. R. Kessener, S. C. Moratti, A. B. Holmes and R. H. Friend, *Chem. Phys. Lett.*, **1995**, *241*, 89–96.
